# The IGFBP3/TMEM219 pathway regulates beta cell homeostasis

**DOI:** 10.1038/s41467-022-28360-2

**Published:** 2022-02-03

**Authors:** Francesca D’Addio, Anna Maestroni, Emma Assi, Moufida Ben Nasr, Giovanni Amabile, Vera Usuelli, Cristian Loretelli, Federico Bertuzzi, Barbara Antonioli, Francesco Cardarelli, Basset El Essawy, Anna Solini, Ivan C. Gerling, Cristina Bianchi, Gabriella Becchi, Serena Mazzucchelli, Domenico Corradi, Gian Paolo Fadini, Diego Foschi, James F. Markmann, Emanuela Orsi, Jan Škrha, Maria Gabriella Camboni, Reza Abdi, A. M. James Shapiro, Franco Folli, Johnny Ludvigsson, Stefano Del Prato, Gianvincenzo Zuccotti, Paolo Fiorina

**Affiliations:** 1 International Center for T1D, Pediatric Clinical Research Center Romeo ed Enrica Invernizzi, DIBIC, Università di Milano, Milan, Italy; 2 Nephrology Division, Boston Children’s Hospital and Transplantation Research Center, Brigham and Women’s Hospital, Harvard Medical School, Boston, MA USA; 3 Enthera S.r.l., Milano, Italy; 4 Diabetology Unit, ASST Grande Ospedale Metropolitano Niguarda, Milan, Italy; 5 NEST-Scuola Normale Superiore, Pisa, Italy; 6 Transplantation Research Center, Nephrology Division, Brigham and Women’s Hospital, Boston, MA USA; 7 Medicine, Al-Azhar University, Cairo, Egypt; 8 Department of Surgical, Medical and Molecular Pathology and Critical Care Medicine, University of Pisa, Pisa, Italy; 9 Department of Medicine, University of Tennessee, Memphis, TN USA; 10 Section of Diabetes and Metabolic Disease, Department of Clinical and Experimental Medicine, University of Pisa and Azienda Ospedaliero-Universitaria Pisana, Pisa, Italy; 11 Department of Medicine and Surgery, Unit of Pathology, University of Parma, Parma, Italy; 12 Department of Medicine, University of Padua, Padua, Italy; 13 General Surgery, DIBIC, L. Sacco Hospital, Università di Milano, Milan, Italy; 14 Division of Transplantation, Department of Surgery, Massachusetts General Hospital, Harvard Medical School, Boston, MA USA; 15 Diabetes Service, Endocrinology and Metabolic Diseases Unit, IRCCS Cà Granda - Ospedale Maggiore Policlinico Foundation, Milan, Italy; 16 3rd Department of Internal Medicine, Charles University, First Faculty of Medicine, Prague, Czech Republic; 17 Clinical Islet Transplant Program, Alberta Diabetes Institute, University of Alberta, Edmonton, AB Canada; 18 Endocrinology and Metabolism, Department of Health Science, Università di Milano, ASST Santi Paolo e Carlo, Milan, Italy; 19 Crown Princess Victoria Children´s Hospital and Div of Pediatrics, Dept of Biomedical and Clinical Sciences, Linköping University, Linköping, Sweden; 20 Pediatric Clinical Research Center Romeo ed Enrica Invernizzi, DIBIC, Università di Milano and Department of Pediatrics, Buzzi Children’s Hospital, Milan, Italy; 21 Division of Endocrinology, ASST Fatebenefratelli-Sacco, Milan, Italy

**Keywords:** Apoptosis, Diabetes, Translational research

## Abstract

Loss of pancreatic beta cells is a central feature of type 1 (T1D) and type 2 (T2D) diabetes, but a therapeutic strategy to preserve beta cell mass remains to be established. Here we show that the death receptor TMEM219 is expressed on pancreatic beta cells and that signaling through its ligand insulin-like growth factor binding protein 3 (IGFBP3) leads to beta cell loss and dysfunction. Increased peripheral IGFBP3 was observed in established and at-risk T1D/T2D patients and was confirmed in T1D/T2D preclinical models, suggesting that dysfunctional IGFBP3/TMEM219 signaling is associated with abnormalities in beta cells homeostasis. In vitro and in vivo short-term IGFBP3/TMEM219 inhibition and TMEM219 genetic ablation preserved beta cells and prevented/delayed diabetes onset, while long-term IGFBP3/TMEM219 blockade allowed for beta cell expansion. Interestingly, in several patients’ cohorts restoration of appropriate IGFBP3 levels was associated with improved beta cell function. The IGFBP3/TMEM219 pathway is thus shown to be a physiological regulator of beta cell homeostasis and is also demonstrated to be disrupted in T1D/T2D. IGFBP3/TMEM219 targeting may therefore serve as a therapeutic option in diabetes.

## Abstract (web summary)

In this new study the Authors demonstrated that the IGFBP3/TMEM219 pathway is a physiological regulator of pancreatic beta cell homeostasis and it is dysregulated in diabetes. IGFBP3/TMEM219 targeting may therefore serve as a therapeutic option in diabetes.

## Introduction

Pancreatic beta-cell mass and homeostasis are primarily preserved and maintained through a finely tuned balance of life and death^1,2^. Several stress factors (e.g., glucolipotoxicity, oxidative stress, and immune attack/infiltration) that impair beta-cell function^3,4^, as well as growth factors and circulating hormones that allow for beta-cell proliferation and renewal^5–9^, have been extensively studied, but little is known with respect to the way(s) by which beta-cell loss can be prevented. Current therapeutics employed in diabetes are now being tested for their potential effects in preserving beta-cell survival, given that maintaining endogenous insulin secretion—even to a minor degree—may improve clinical outcomes in diabetic patients^10,11^. Moreover, a growing body of evidence suggests a substantial beneficial effect in specifically targeting beta-cell death as a key mechanism to preserve the beta-cell pool and prevent or delay diabetes onset^12–15^. TMEM219 is a recently discovered death receptor, which induces cell apoptosis by a Caspase-8-dependent mechanism, through the binding with its ligand, the Insulin-like growth factors binding protein 3 (IGFBP3)^16,17^. Indeed, IGFBP3 acts as a carrier of nearly 95% of insulin-like growth factor-1 (IGF-I) and IGF-II in the circulation^18^, but it also has an IGF-I independent effect on TMEM219-expressing intestinal stem cells^19,20^. The crucial role of the IGFBP3 signaling in modulating cell fate and tissue growth has been also supported in preclinical models in which a reduced islet mass followed by dysglycemia was observed in the presence of constitutive overexpression of IGFBP3^21^. In this study, we hypothesize that IGFBP3 acts as a new regulator of beta-cell mass by binding the death receptor TMEM219, which we demonstrate here is expressed in islet beta cells, thus promoting beta-cell loss. Pharmacological or genetic targeting of the IGFBP3/TMEM219 pathway successfully protects beta-cell mass, facilitates beta-cell expansion, and prevents and delays diabetes onset, suggesting a new therapeutic option for diabetic patients.

## Results

### The death receptor TMEM219 is expressed in islet beta cells

**Fig. 1:**
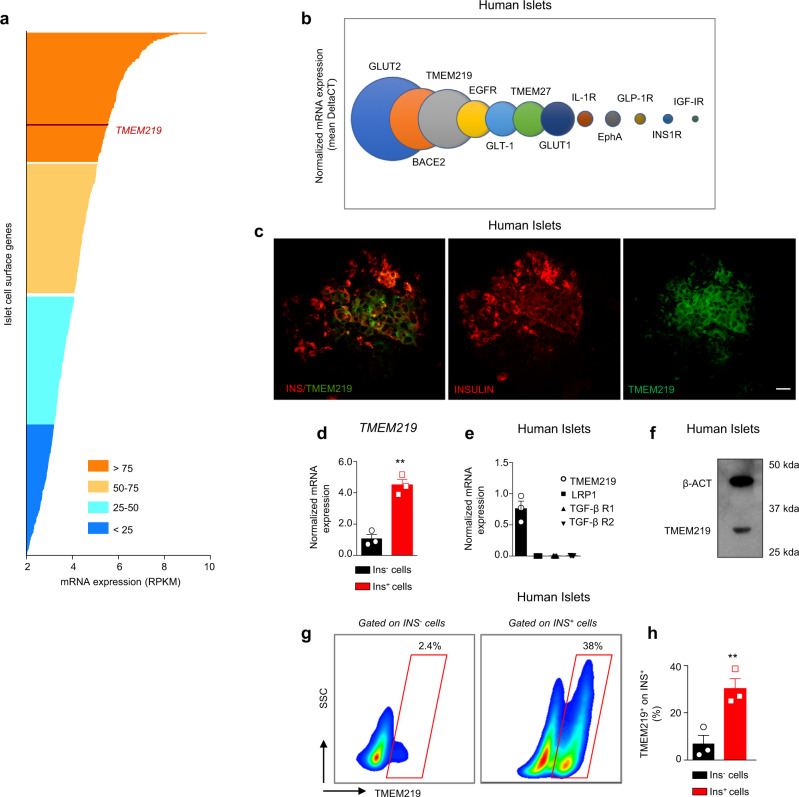
The death receptor TMEM219 is expressed in islet beta cells. **a** The transcriptome profile of genes encoding islet surface proteins was screened using RNA-seq of islets isolated from non-diabetic donors (*n* = 4). A full list of genes analyzed is reported in Supplementary Data 2. **b** Quantification of *TMEM219* relative expression as compared to other relevant receptors in human islets by targeted qRT-PCR (*n* = 4). **c** Representative image of TMEM219 (green) and INS (red) co-expression in purified human islets of non-diabetic donors. Original magnification ×40, scale bar 25 μm. Merge is in the left panel. **d** Bar graph representing the expression of TMEM219 mRNA on flow-sorted insulin-positive and -negative cells obtained from purified human islets isolated from non-diabetic donors (*n* = 3). **e** Bar graphs comparing expression of *TMEM219* and other IGFBP3 putative receptors (*LRP1*, *TGF-β R1*, and *TGF-β R2*) analyzed by qRT-PCR in purified human islets (*n* = 3). **f** Representative immunoblot of TMEM219 protein expression in human islets (with β-actin as a control). **g**, **h** Representative flow plot and a quantitative bar graph showing TMEM219 expression in insulin-positive and -negative cells detected using flow cytometry in healthy human islets (*n* = 3). Data are expressed as mean ± standard error of the mean (SEM) unless otherwise reported. **P < 0.01 by two-sided *t* test. mRNA expression was normalized to β-actin (*ACTB*). Experiments were performed in duplicate. The data shown in (**c**) and in (**f**) are the representative result from three independent experiments. Source data are provided as a Source Data file. RNAseq RNA sequencing analysis, RPKM reads per kilobase per million, INS insulin, qRT-PCR quantitative real-time polymerase chain reaction.

To identify novel pathways potentially involved in beta cells homeostasis, we performed transcriptome profiling in purified human islets of non-diabetic donors with a particular focus on islet surface proteins. The death receptor TMEM219 was one of the most highly expressed (Fig. 1a) and ranked the third most abundant beta-cell receptor among all those subsequently quantified by targeted qRT-PCR analysis (Fig. 1b). Expression of TMEM219 in purified human islets was further confirmed by confocal imaging (Fig. 1c), with flow-sorted islet-derived insulin-positive cells expressing higher TMEM219 mRNA as compared to islet-derived insulin-negative cells (Fig. 1d). Interestingly, *TMEM219* was the sole IGFBP3 receptor expressed on beta cells, as the mRNA expression of other putative IGFBP3 receptors (e.g., low-density lipoprotein receptor-related protein 1, *LRP1*, *TGF-β Type 1*, and *Type 2 Receptor*) was undetectable (Fig. 1e). Moreover, TMEM219 expression was also evident by immunoblotting (Fig. 1f), by immunohistochemistry (Supplementary Fig. 1a), and flow cytometry (Fig. 1g) analysis, the latter performed on insulin-positive cells (Supplementary Fig. 1b) and confirming a higher percentage of TMEM219^+^ cells within the insulin-positive fraction (Fig. 1g, h). We next analyzed two human immortalized beta-cell lines and human alpha cells and showed that TMEM219 protein and mRNA are barely detectable on alpha cells, with nearly threefold and 80-fold increased expression in beta cells (Supplementary Fig. 1c–f), where colocalization with insulin was also evident by confocal imaging analysis (Supplementary Fig. 1g).

### Peripheral levels of the TMEM219 ligand IGFBP3 are altered in diabetic patients

**Fig. 2:**
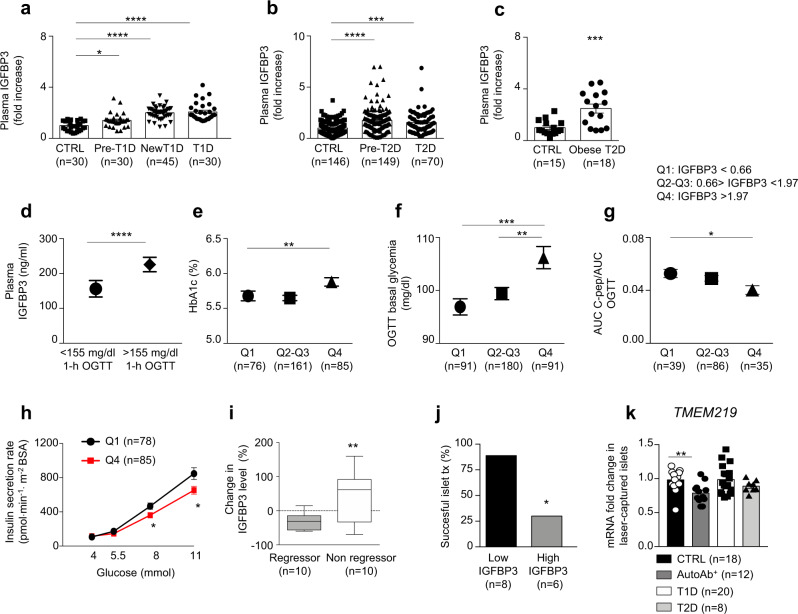
Peripheral levels of TMEM219 ligand IGFBP3 are altered in diabetic patients. **a** Scatter plots representing fold increase in peripheral IGFBP3 in patients with established T1D (*n* = 30), with new-onset T1D (*n* = 45), at risk for developing T1D (with at least one detected autoantibody, pre-T1D, *n* = 30), as compared to non-diabetic healthy controls (CTRL, *n* = 30). Demographic characteristics are reported in Supplementary Table 1. **b** Scatter plots representing fold increase in peripheral IGFBP3 patients with newly diagnosed T2D (*n* = 70), at risk for developing T2D (with altered glucose tolerance, pre-T2D, *n* = 149) as compared to non-diabetic healthy controls (with normal glucose tolerance, NGT, *n* = 146). Demographic characteristics are reported in Supplementary Table 2. **c** Box plot showing fold increase in peripheral IGFBP3 in obese T2D patients undergoing bariatric surgery (*n* = 18, white) as compared to non-diabetic healthy controls (*n* = 15, black). **d** Bar graph representing mean ± SEM peripheral IGFBP3 in patients with 1- h blood glucose level >155 mg/dl (*n* = 256) at the oral glucose tolerance test (OGTT) as compared to those with a 1-h blood glucose level <155 mg/dl (*n* = 105), within the cohort shown in panel **b** (NGT, pre-T2D and T2D groups). **e**, **f**, **g** Single scatter plot representing mean ± SEM percentage of HbA1c (**e**), OGTT basal glycemia (**f**) and C-peptide AUC/OGTT AUC ratio (**g**) measured in quartiles subgroups of IGFBP3 showed in panel **b** (NGT, pre-T2D, and T2D groups) and established as following: <0.66 (Q1), ranging between 0.66–1.97 (Q2-Q3) and >1.97 (Q4). (**h**). Line graph comparing insulin-secretion rate measured at different glucose concentrations (4, 5.5, 8, and 11 mM) in Q1 and Q4 IGFBP3 quartiles subgroups shown in panel **b** (NGT, pre-T2D and T2D groups). **i** Box plot depicting percent change in peripheral IGFBP3 levels measured in diabetic patients who underwent liver transplantation and who near-normalized (gray) glycometabolic control after transplantation (regressor) or remained diabetic (white, non-regressor). All box plots include the median line, the whiskers indicate the minimum and maximum value and the box of the box plot illustrates the upper and lower quartile (two-sided *t* test). Demographic characteristics are reported in Supplementary Table 3. **j** Bar graph representing the percentage of patients with successful islet transplantation (C-peptide >0.4 ng/ml) who displayed low (<1.92, Q1–Q2) vs. high (>1.92, Q3–Q4) peripheral IGFBP3 fold changes. **k** Bar graph representing TMEM219 mRNA expression by RNAseq in laser-captured islets obtained from human non-diabetic subjects (CTRL, *n* = 18), individuals with T1D (*n* = 20), individuals at risk for T1D (AutoAb^+^, *n* = 12) and individuals with T2D (*n* = 8). mRNA expression was expressed as fold change as compared to CTRL. Fold change was computed as the ratio of the changes between T1D, AutoAb+ and T2D values and the CTRL value over the mean CTRL value. Data are expressed as mean ± standard error of the mean (SEM) unless otherwise reported. *P < 0.05; **P < 0.01; ***P < 0.001, ****P < 0.0001 by Kruskal–Wallis adjusted for multiple comparison, Mann–Whitney *U* test, one-way ANOVA followed by Bonferroni post hoc test, two-way ANOVA, two-sided *t* test and Chi-square test. Source data are provided as a Source Data file. CTRL healthy volunteers, T1D type 1 diabetes, pre-T1D patients showing positive results for at least one autoantibody, AutoAb autoantibodies, T2D type 2 diabetes, NGT normal glucose tolerance, IGT impaired glucose tolerance, New T1D new-onset T1D, Q quartile, vs versus, qRT-PCR quantitative real-time polymerase chain reaction.

As TMEM219 is expressed on islet beta cells, we next assessed whether peripheral levels of TMEM219 ligand IGFBP3 were altered in patients with diabetes. Of note, we observed increased peripheral IGFBP3 in patients with established T1D, in those with new-onset T1D, and, although to a lesser extent, in those at risk for developing the disease (based on the detection of one or more anti-islet autoantibodies) as compared to non-diabetic healthy volunteers (Fig. 2a and Supplementary Table 1). This observation was paralleled in patients with newly diagnosed T2D and in those with impaired glucose tolerance, as compared to patients with normal glucose tolerance (Fig. 2b and Supplementary Table 2), and further confirmed in obese diabetic patients selected to undergo bariatric surgery (Fig. 2c). Interestingly, the analysis of glycometabolic parameters in a cohort of individuals at risk for T2D and enrolled within a subgroup of the GENFIEV study revealed that peripheral IGFBP3 levels were higher in patients with 1-h OGTT (oral glucose tolerance test) plasma glucose level >155 mg/dl, which is a strong predictor of T2D development (Fig. 2d). By further dividing fold changes of IGFBP3 observed in this population into quartiles, we demonstrated that elevated IGFBP3 levels were associated with higher HbA1c, higher fasting plasma-glucose levels, lower insulin sensitivity, and lower insulin-secretion rate (Fig. 2e–h), thus supporting the association between dysregulated IGFBP3 and beta-cell dysfunction. Indeed, peripheral levels of insulin-like growth factor I (IGF-I), of which IGFBP3 is the main circulating carrier, were altered only to some extent in diabetic and pre-diabetic conditions (Supplementary Fig. 2a, b), thus suggesting a greater increase in IGFBP3 as compared to IGF-I, as shown also by the IGFBP3/IGF-I ratio (Supplementary Fig. 2c, d). This was also supported by demonstrating that plasma levels of IGF-II, which is also carried by IGFBP3 in the circulation, were comparable in diabetic and non-diabetic subjects, and by further confirming increased peripheral IGFBP3 in diabetes through Luminex technology (Supplementary Fig. 2e–h) and by validating the analysis through a clinically grade electrochemiluminescence immune-assay (Supplementary Fig. 2i). The dysregulation of the IGFBP3/TMEM219 axis was confirmed in two additional highly relevant cohorts of patients (Supplementary Tables 3 and 4): (i) diabetic patients undergoing liver transplantation (Fig. 2i and Supplementary Table 3), in which those who near-normalized their glycometabolic control after transplantation (regressor) showed a significant reduction in peripheral IGFBP3 as compared to those who remained diabetic (non-regressor); and (ii) islet-transplanted patients, in which partially successful islet transplantation in T1D (i.e., achieving C-peptide higher than 0.4 ng/ml after transplant) was associated with lower peripheral IGFBP3 levels as compared to higher IGFBP3 levels observed in failing islet transplantation (Fig. 2j). In order to understand the basis for increased production of IGFBP3, which is primarily released by the liver, we cultured a human hepatocytes-derived cell line (Huh7) in vitro with sera of diabetic T1D or T2D patients in place of regular FBS and demonstrated increased secretion of IGFBP3 as compared to IGFBP3 levels measured in the culture of hepatocytes exposed to sera obtained from healthy volunteers (CTRL) or to high-glucose medium (Supplementary Fig. 2j). Elevated IGFBP3 hepatocytes production was also obtained during a challenge with a diabetogenic pro-inflammatory milieu (IL-1β + /− IFN-γ, Supplementary Fig. 2k). Interestingly, immune neutralization of IL-1β and/or IFN-γ fully abrogated the increased IGFBP3 hepatic production observed upon T1D/T2D sera exposure (Supplementary Fig. 2j). Given the altered IGFBP3 levels observed in diabetic patients, we interrogated TMEM219 expression in laser-captured islets through RNA sequencing and confirmed it in healthy subjects, patients with T1D, with T2D or at risk for T1D, without any difference among groups (Fig. 2k). In summary, these data suggest that a dysfunctional IGFBP3/TMEM219 pathway is associated with a disrupted beta-cell homeostasis.

### Role of IGFBP3/TMEM219 signaling in islet and beta-cell apoptosis

**Fig. 3:**
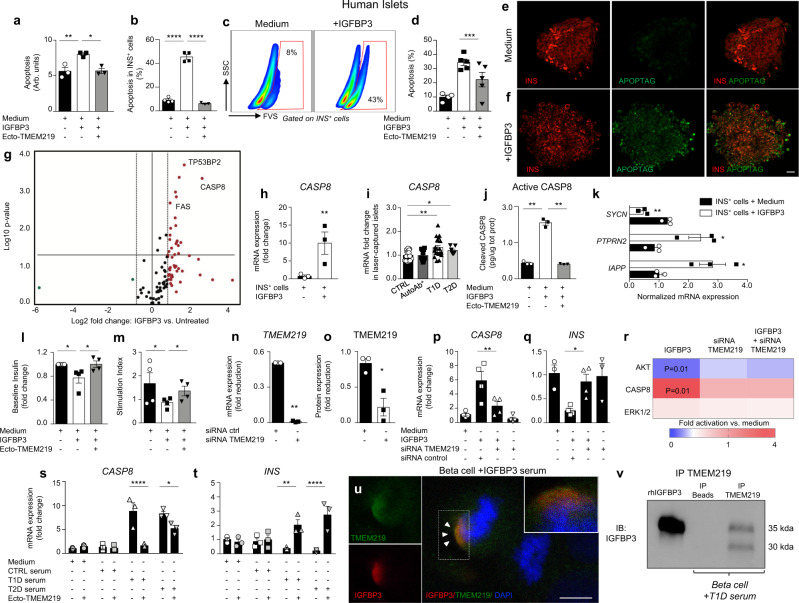
IGFBP3/TMEM219 signaling triggers beta-cell apoptosis. **a** Bar graph quantifying apoptosis in human islets cultured with/without IGFBP3 and in the presence/absence of ecto-TMEM219 (*n* = 3). The experiment was run in duplicate. **b**, **c** Bar graph and representative flow plots quantifying cell death of insulin-positive cells in human islets cultured with/without IGFBP3 and in the presence/absence of ecto-TMEM219 (*n* = 3). **d** Bar graph (percentage of double-positive cells analyzed per field) depicting apoptosis (green, Apoptag) of insulin-positive (red) cells in human purified islets cultured with/without IGFBP3 and in the presence/absence of ecto-TMEM219 (*n* = 5). **e**, **f** Anecdotical picture of INS (red, left panels) and APOPTAG (green, middle panels) immunofluorescence in purified human islets of non-diabetic donors cultured with/without IGFBP3. Merge images are in right panels. Original magnification ×40, scale bar 25 μm. **g** Scatter plot representing the transcriptome profile of relevant-apoptotic genes in human islets cultured with/without IGFBP3 (*n* = 3). The experiment was run in triplicate. The complete dataset of differentially expressed genes is reported in Supplementary Table 5. **h** Bar graph representing normalized mRNA expression of *CASP8* quantified by qRT-PCR in flow-sorted insulin-positive cells obtained from human islets cultured with/without IGFBP3 (*n* = 3). **i** Bar graph representing CASP8 mRNA expression quantified by RNAseq in laser-captured islets obtained from human non-diabetic subjects (CTRL, *n* = 18), patients with T1D (*n* = 20), patients at risk for T1D (AutoAb^+^, *n* = 12) and patients with T2D (*n* = 8). mRNA expression is shown as fold change as compared to CTRL. **j** Bar graph quantifying Cleaved Caspase 8 by ELISA in human islets cultured with/without IGFBP3 and in the presence/absence of ecto-TMEM219 (*n* = 3). **k** Grouped graph depicting targeted gene expression analysis of insulin secretory machinery using qRT-PCR in insulin-positive cells flow-sorted from human purified islets (*n* = 3) cultured with/without IGFBP3. mRNA was normalized to *ACTB*, and the experiment was performed in triplicate. **l** Bar graph depicting baseline insulin secretion measured in the supernatant of human islets cultured with/without IGFBP3 and in the presence or absence of ecto-TMEM219 (*n* = 4). **m** Bar graph showing the stimulation index calculated by measuring insulin secretion in the supernatant of human islets upon glucose stimulation and cultured with/without IGFBP3 and in the presence or absence of ecto-TMEM219 (*n* = 4). **n**, **o** Bar graphs confirming the absence of TMEM219 mRNA (**n**) and protein (**o**) expression upon transient silencing in human islets (*n* = 3). The experiment was performed in duplicate. **p**, **q** Bar graphs depicting normalized expression (fold change) of *CASP8* (**p**) and *INS* (**q**) quantified by qRT-PCR in purified human islets, in which TMEM219 was targeted via siRNA delivery, compared to islets in which TMEM219 targeting was not performed (*n* = 4 samples per group of treatment). **r** Heatmap showing fold activation of AKT, Caspase 8, and ERK1/2 in human islets cultured with/without IGFBP3 and/or in which TMEM219 was targeted via siRNA delivery (*n* = 3 samples/group). Fold activation was assessed by immunoblotting and normalized to that measured in medium-cultured islets. **s**, **t** Bar graphs depicting normalized expression (fold change) of *CASP8* (**s**) and *INS* (**t**) quantified by qRT-PCR in purified human islets exposed to medium or to sera of CTRL, T1D, and T2D patients (pooled from *n* = 5 donors per group) and in the presence/absence of ecto-TMEM219 (*n* = 3 islet preparations per group of treatment). Experiments were performed in duplicate. **u** Representative picture of confocal microscopy analysis (scale bar 10 μm, ×63 original magnification) depicting colocalization of TMEM219 (green) and serum IGFBP3 (red) in a human beta-cell line (Blox-5) cultured with T1D serum. Cells were stained with DAPI (blue) and immunolabeled with anti-TMEM219 (green) and anti-IGFP3 Abs (red). **v** Representative blot showing TMEM219 immunoprecipitation (IP) in human beta-cell line (Blox-5) cultured with serum naturally enriched in IGFBP3 (T1D serum) pooled from *n* = 5 donors. Expression of IGFBP3 is shown. Lane 1: rh-IGFBP3. Lane 2: IP with beads alone. Lane 3: IP with TMEM219 Ab. Data are expressed as mean ± standard error of the mean (SEM) unless otherwise reported. *P < 0.05; **P < 0.01; ***P < 0.001; ****P < 0.0001 by one-way ANOVA followed by Sidak/Bonferroni’s post hoc test, two-sided *t* test, Kruskal–Wallis test adjusted for multiple comparisons. mRNA expression was normalized to *ACTB*. The data shown in (**e**, **f**, **u**, **v**) are the representative result from three independent experiments. Source data are provided as a Source Data file. T1D type 1 diabetes, T2D type 2 diabetes, AutoAb^+^ positive for autoantibodies, *INS* insulin, *CASP8* Caspase 8, siRNA small interfering RNA, ecto-TMEM219 newly generated recombinant protein based on TMEM219 extracellular portion, Arb. Units arbitrary units.

To next demonstrate the effect of IGFBP3-mediated TMEM219 signaling in pancreatic beta cells, we first challenged purified human islets obtained from healthy subjects and not suitable for donation, with IGFBP3 in vitro and observed a higher rate of apoptosis/cell death (Fig. 3a). Next, we demonstrated by flow cytometry and immunostaining that this IGFBP3-mediated detrimental effect is evident mainly in insulin-positive cells (Fig. 3b, c, d, e, f). A transcriptome profiling conducted on whole islets showed a marked upregulation of pro-apoptotic genes (i.e., *CASP8*, *CASP9*, *TP53BP2*, *TNFSF10*, *FAS*) in IGFBP3-challenged islets as compared to untreated islets (Fig. 3g, Supplementary Table 5), with Caspase 8 observed to be the most highly abundant when its relative expression was compared to other pro-apoptotic genes. Indeed, Caspase-8 expression was confirmed to be upregulated also in flow-sorted human islet insulin-positive cells during IGFBP3 challenge in vitro (Fig. 3h) and in laser-captured islets of T1D and T2D patients as compared to those of healthy subjects (Fig. 3i). The upregulated Caspase-8 mRNA expression was paralleled by an increase in cleaved Caspase 8 in IGFBP3-cultured islets and confirmed an IGFBP3-mediated Caspase-8 activation (Fig. 3j). Interestingly, flow-sorted human islet insulin-positive cells also showed an alteration in the expression of the insulin secretory machinery-related genes *SYMC*, *IAPP*, and *PTPRN2*, during IGFBP3 challenge in vitro (Fig. 3k), which was associated with a dysregulation in insulin granules size and number in purified human islets and motility in beta-cell line (Supplementary Fig. 3a, b). A decreased insulin secretion at baseline and upon glucose stimulation in IGFBP3-cultured human islets further demonstrated the detrimental effect of IGFBP3 in beta-cell function (Fig. 3l, m). Indeed, our transcriptome analysis also showed that insulin gene expression (*INS*) was lowest in its abundance amongst all beta-cell-related genes in IGFBP3-cultured islets, and that expression of transcripts linked to insulin signaling/secretion (e.g., *IRS1*, *MAPK1*, *FBP1*, *PPTN1*) was significantly altered (Supplementary Table 6). To next demonstrate that IGFBP3-mediated apoptosis of beta cells was triggered through the activation of TMEM219 death signaling in beta cells, we targeted TMEM219 in human islets using siRNA (Fig. 3n, o) and observed that Caspase 8 and insulin expression were preserved despite the addition of IGFBP3 (Fig. 3p, q). Interestingly, silencing of TMEM219 also abrogated the IGFBP3-mediated effect on Caspase 8 and re-established the physiological AKT activation, which is involved in preserving cell survival and was blocked when IGFBP3 was added to the culture (Fig. 3r and Supplementary Fig. 3c). Conversely, no effect was evident on another IGFBP3-related pathway, which finely controls cell proliferation/death, the ERK1/2 (Fig. 3r and Supplementary Fig. 3d, e), thus suggesting that Caspase-8 activation and AKT-blockade are engaged in the IGFBP3/TMEM219-dependent cell death/apoptosis signaling cascade. Finally, blockade of IGF-I/IGF-IR did not prevent IGFBP3-mediated islet apoptosis in vitro, thus suggesting that IGFBP3 is not acting solely by blocking the survival effect of ambient IGFs (Supplementary Fig. 3f). To further determine whether higher peripheral IGFBP3 levels favor islet injury in disease conditions such as diabetes, we challenged human islets with sera obtained from patients with either T1D or T2D, which are naturally enriched in IGFBP3 as compared to those of healthy subjects, in place of regular FBS, and confirmed increased Caspase 8 and decreased insulin mRNA expression (Fig. 3s, t). To better address the engagement of IGFBP3 as circulating in blood with TMEM219 expressed in target cells, we first demonstrated, through analysis of immunofluorescence distribution at the single-cell level, a colocalization between serum IGFBP3 and TMEM219 in beta cells cultured in vitro with T1D serum, which was not evident in those cultured with non-diabetic serum or with FBS (Fig. 3u and Supplementary Fig. 3g, h). Next, TMEM219-expressing beta cells cultured with T1D serum, were subjected to immunoprecipitation with anti-TMEM219 antibody and the immunoblotting analysis of the precipitate confirmed that TMEM219 interacts with serum IGFBP3 (Fig. 3v). We finally conducted a series of experiments in which a recombinant protein, ecto-TMEM219, which we have generated by cloning the extracellular domain of TMEM219 receptor and acts by preventing the IGFBP3/TMEM219 binding, was used to pharmacologically block the IGFBP3/TMEM219 pathway. Interestingly, the pro-apoptotic Caspase-8-associated effect and reduction in insulin expression and release induced by IGFBP3 exposure in vitro were rescued by ecto-TMEM219 in purified human islets (Fig. 3a, b, d, j and Supplementary Fig. 3i, j). Ecto-TMEM219 was also able to re-establish the physiological expression of Caspase 8 and insulin mRNA in purified human islets upon culture with serum of either T1D or T2D patients in place of regular FBS, and as compared with the serum of healthy subjects (Fig. 3s, t). Lastly, all the aforementioned experiments performed in a human beta-cell line (Blox-5) mechanistically confirmed the effects of the IGFBP3/TMEM219 pathway and of Ecto-TMEM219 pharmacological blockade on beta cells by faithfully replicating our observations in human islets (Supplementary Fig. 4). The dose-effect correlation between Caspase 8 and cell death upon IGFBP3 exposure (Supplementary Fig. 4a–c) and the abrogation of the Caspase-8-associated beta-cell apoptosis obtained through the depletion of IGFBP3 from the T1D/T2D serum or with the use of an anti-IGFBP3 monoclonal antibody during the serum challenge of beta cells, further reinforced our findings (Supplementary Fig. 4d–f). The abrogation of Caspase-8 activation with a selective Caspase-8 inhibitor, despite some experimental limitations^22^, and through TMEM219 silencing by siRNA (Supplementary Fig. 4g), also supported the IGFBP3-mediated Caspase-8 effect in beta cells. In summary, we demonstrated that the IGFBP3/TMEM219 signaling is deleterious for beta cells via a Caspase-8 activation/AKT inhibition mechanism, and that IGFBP3/TMEM219 blockade may prevent beta-cell death/dysfunction (Supplementary Fig. 4h).

### Targeting IGFBP3/TMEM219 signaling protects beta cells in preclinical models of diabetes in vivo

**Fig. 4:**
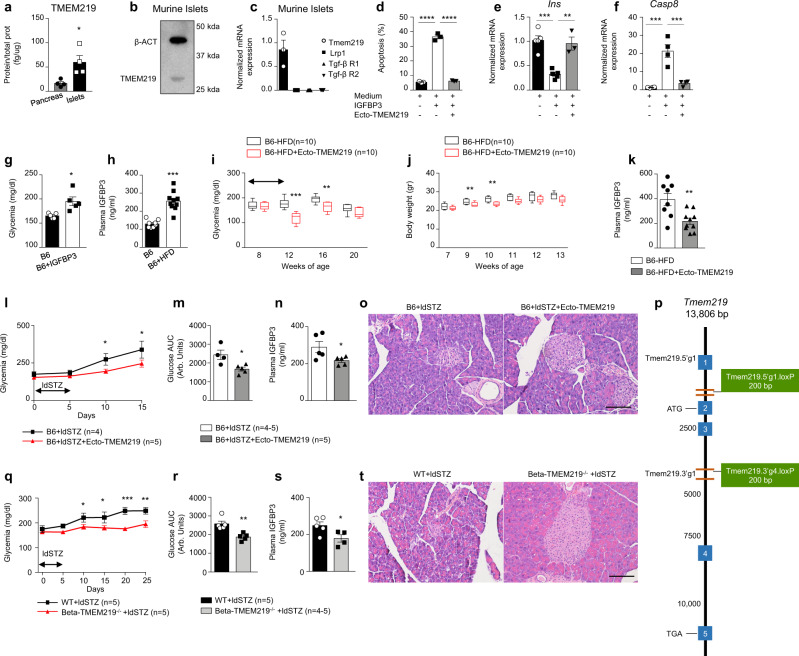
Targeting IGFBP3/TMEM219 signaling protects beta cells in preclinical models of diabetes in vivo. **a** Bar graph showing TMEM219 protein expression (ELISA) assessed in the whole pancreas and dissociated pancreatic islets obtained from B6 mice (*n* = 4). The experiment was performed in duplicate. **b** Representative immunoblot of TMEM219 protein expression in murine islets (β-actin was used as a control, *n* = 3). **c** Bar graph comparing *Tmem219* and other IGFBP3 receptors (*Lrp1*, *Tgf-β R1*, and *R2*) gene expression quantified by qRT-PCR in purified murine islets of B6 mice (*n* = 3). **d** Quantitative bar graph (percentage of double-positive cells analyzed per field) depicting apoptosis in purified murine (B6) islets cultured with/without IGFBP3 in the presence or absence of ecto-TMEM219 (*n* = 3). **e**, **f** Bar graphs representing *Ins* (**e**) and *Casp8* (**f**) mRNA expression analyzed by qRT-PCR in murine islets cultured with/without IGFBP3 and in the presence or not of ecto-TMEM219 (*n* = 4 in (**f**) and *n* = 5 in (**e**)). The experiment was performed in duplicate. **g** Bar graph depicting fasting blood glucose levels measured in IGFBP3-treated and untreated B6 mice (*n* = 5/group, day 7). (**h**). Bar graph depicting peripheral IGFBP3 levels measured in plasma obtained from 8-week-old B6 mice fed a high-fat diet (B6-HFD, *n* = 10) or left untreated (B6, *n* = 8). **i** Box plots representing blood glucose levels measured in B6 mice fed a high-fat diet (B6-HFD) treated with (red lines)/without (black lines) ecto-TMEM219 (*n* = 8–10/group). **j** Box plots representing body weight measured in B6 mice fed a high-fat diet (B6-HFD) treated with (red dots)/without (black dots) ecto-TMEM219 (*n* = 8–10/group). In (**i**) and (**j**) box plots include the median line, the whiskers indicate the minimum and maximum value, and the box of the box plot illustrates the upper and lower quartile. **k** Bar graph representing peripheral IGFBP3 levels in ecto-TMEM219-treated and untreated B6-HFD mice (*n* = 8 and 10, respectively). **l** Line graph showing blood glucose level measured in B6 mice injected with multiple low-dose of streptozotocin (ldSTZ, 50 mg/Kg) and treated with ecto-TMEM219 or left untreated (*n* = 5). **m** Bar graph showing blood glucose area under the curve (AUC) measured at the intraperitoneal glucose (1 g/Kg) tolerance test (IPGTT) in B6 mice injected with multiple low-dose of streptozotocin and treated with ecto-TMEM219 or left untreated at day 10 (*n* = 5 and 4, respectively). **n** Bar graph showing IGFP3 plasma levels measured in B6 mice injected with multiple low-dose of streptozotocin and treated with ecto-TMEM219 or left untreated at day 10 (*n* = 5). **o** Representative H&E staining in serial pancreatic islet tissue sections obtained from B6 mice injected with multiple low dose of streptozotocin and treated with ecto-TMEM219 or left untreated (*n* = 3/group). ×20 original magnification, scale bar, 100 μm. **p** Targeting strategy to generate the TMEM219^fl/fl^ mouse by using the Cre-*loxP* strategy. **q** Line graph showing blood glucose level measured in Beta-TMEM219^−/−^ and in wild-type (WT) mice injected with multiple low-dose of streptozotocin (*n* = 5). **r** Bar graph showing blood glucose area under the curve (AUC) measured at the IPGTT in Beta-TMEM219^−/−^ and in wild type (WT) mice injected with multiple low-dose of streptozotocin (*n* = 5). **s** Bar graph showing IGFP3 plasma levels measured in Beta-TMEM219^−/−^ and in wild-type (WT) mice injected with multiple low doses of streptozotocin (*n* = 4 and 5, respectively). **t** Representative H&E staining in serial pancreatic islet tissue sections obtained from WT and Beta-TMEM219^−/−^ injected with multiple low dose of streptozotocin (*n* = 3/group). ×20 original magnification, scale bar, 100 μm. In (**i**, **j**, **l**, **q**) statistical analysis compared blood glucose levels or weight expressed as mean ± SEM (**l**, **q**) between the two groups at each timepoint. Data are expressed as mean ± standard error of the mean (SEM) unless otherwise reported. *P < 0.05; **P < 0.01; ***P < 0.001; ****P < 0.0001 by one-way ANOVA followed by Bonferroni’s post hoc test, two-way ANOVA, two-sided Mann–Whitney *U* test or two-sided *t* test. mRNA expression was normalized to *Gapdh*. The data shown in o1–o2, t1–t2 are the representative result from three independent experiments. Source data are provided as a Source Data file. B6 C57BL/6J mice, HFD high-fat diet, hyper hyperglycemic, mAb monoclonal antibody, Arb. units arbitrary units, Beta-TMEM219^−/−^ mice in which TMEM219 was deleted in beta cells, WT wild-type mice in which TMEM219 has not been genetically deleted, STZ streptozotocin, ldSTZ low-dose streptozotocin 50 mg/Kg injected for 5 days.

In order to assess the relevance of the IGFBP3/TMEM219 axis and its blockade in preserving the beta-cell homeostasis in vivo in preclinical models, we first demonstrated that TMEM219 is expressed in murine pancreas and purified islets of C57BL/6J (B6) mice (Fig. 4a, b). TMEM219 protein and mRNA expression were also shown by immunoblotting and qRT-PCR, and the lack of expression of other IGFBP3 receptors within murine pancreatic islets was further confirmed (Fig. 4b, c). In vitro experiments also showed that the addition of IGFBP3 to purified islets of B6 mice was associated with increased apoptosis, upregulated Caspase 8, and decreased insulin expression (Fig. 4d–f). Interestingly, blockade of the IGFBP3/TMEM219 pathway in vitro with ecto-TMEM219 abrogated the IGFBP3-induced detrimental effects in murine pancreatic islets (Fig. 4d–f). To further confirm our observation in vivo, we first injected a short course of IGFBP3 (0.2 mg daily for 5 days i.p.) in naive B6 mice and observed increased fasting blood glucose levels as compared to untreated controls (Fig. 4g). We therefore interrogated the role of the IGFBP3/TMEM219 axis and its targeting in well-renown in vivo models of diabetes. First, in parallel with our observations in human patients, we demonstrated elevated peripheral IGFBP3 levels in B6 mice receiving or not a high-fat diet (HFD, Fig. 4h), which recapitulates in vivo the features of T2D. As observed in human subjects, peripheral IGF-I levels were not altered in pre-diabetic and diabetic mice (Supplementary Fig. 5a), resulting in an elevated IGFBP3/IGF-I ratio (Supplementary Fig. 5b) that further confirmed the availability of a peripheral IGFBP3 fraction capable of binding TMEM219 on islet cells. We next tested the effect of IGFBP3/TMEM219 pharmacological blockade in the aforementioned preclinical T2D model and demonstrated that administration of the newly generated ecto-TMEM219 significantly reduced blood glucose levels and body weight when given to HFD-treated mice as compared to untreated controls (Fig. 4i, j), and also showed reduced peripheral IGFBP3 levels (Fig. 4k) and increased insulin level, while IGF-I level remained unaltered (Supplementary Fig. 5c, d). In a second model of beta-cell destruction and diabetes, multiple low doses of streptozotocin (ldSTZ, 50 mg/Kg for 5 days), which induces beta-cell death such as that observed in diabetes, was administered to B6 mice. Interestingly, ecto-TMEM219 treatment successfully preserved blood glucose levels in treated mice as compared to untreated animals over time (Fig. 4l), with an improvement also of the glucose AUC at the intraperitoneal glucose tolerance test (IPGTT), (Fig. 4m and Supplementary Fig. 5e) with no effect on body weight and fasting plasma insulin levels (Supplementary Fig. 5f, g). Reduced IGFBP3 plasma levels and preserved islet morphology further confirmed the beneficial effect of ecto-TMEM219-mediated IGFBP3 inhibition in this diabetes model (Fig. 4n, o). To mechanistically link the preservation of beta cells with inhibition of IGFBP3/TMEM219 signaling, we generated a TMEM219^flox/flox^ mouse (Fig. 4p and Supplementary Fig. 6a) and crossed this mouse with the INS^cre^ mouse model to obtain the Beta-TMEM219^−/−^ mouse, in which TMEM219 was conditionally deleted in pancreatic beta cells (Supplementary Fig. 6b, c). While peripheral insulin levels were comparable in Beta-TMEM219^−/−^ mice and wild-type (WT) mice, a decrease in plasma IGFBP3 levels was evident (Supplementary Fig. 6d, e), thus suggesting a dysregulated IGFBP3/TMEM219 axis in this mouse model. Interestingly, the IPGTT revealed a slight improvement of the AUC glucose level in the Beta-TMEM219^−/−^ as compared to WT mice, particularly at the 60 min peak, paralleled by an increased insulin level (Supplementary Fig. 6f–h). To further demonstrate the role of TMEM219 genetic targeting in beta cells, Beta-TMEM219^−/−^ mice were challenged with a multiple low-dose of streptozotocin and showed significantly lower blood glucose level over time as compared to WT mice (Fig. 4q). Notably, low-dose-streptozotocin injected Beta-TMEM219^−/−^ mice demonstrated an improvement in the IPGTT glucose AUC (Fig. 4r), both at the 30 and 60 min time points (Supplementary Fig. 6i), a reduced IGFBP3 plasma level (Fig. 4s), and a preserved islet morphology (Fig. 4t). The aforementioned data confirmed the protective effect exerted by IGFBP3/TMEM219 pharmacologic targeting and TMEM219 genetic ablation in vivo on beta cells.

### Targeting IGFBP3/TMEM219 signaling protects beta cells in the NOD mouse in vivo

**Fig. 5:**
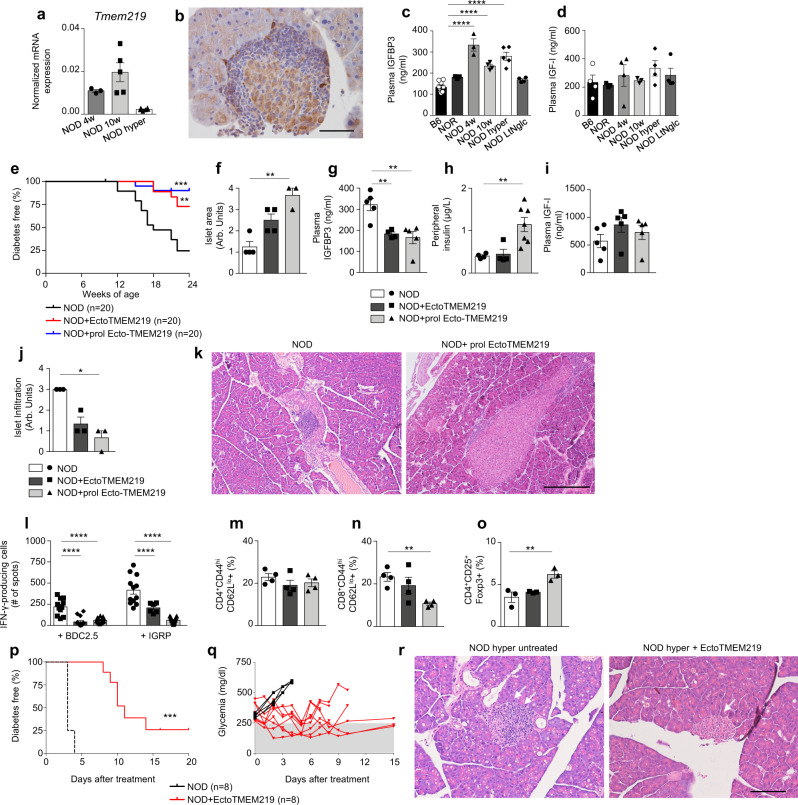
Targeting IGFBP3/TMEM219 signaling protects beta cells in the NOD mouse in vivo. **a** Bar graph depicting Tmem219 mRNA expression analyzed by qRT-PCR in pancreata of NOD mice (*n* = 3, 5, and 4 per group). **b** Representative immunohistochemical TMEM219 staining in serial pancreatic islet tissue sections from NOD mice (*n* = 3). ×40 original magnification, scale bar, 50 μm. **c**, **d** Bar graph depicting peripheral IGFBP3 (**c**) and IGF-I (**d**) levels measured in plasma obtained from 8-week-old B6 (*n* = 10) mice, in pre-diabetic (4-week and 10-week-old, *n* = 4) and diabetic (hyperglycemic, *n* = 5) NOD mice, in mice resistant to the development of autoimmune diabetes (NOR, *n* = 3) and in long-term normoglycemic (LtNglc, 26-week-old, *n* = 4) NOD mice. **e** Ten-week-old NOD mice were treated with ecto-TMEM219 (0.1 mg/mouse/day for 10 days or 0.1 mg/mouse/day for 10 days and twice per week or were left untreated (*n* = 20/group), and the incidence of diabetes was then compared using the log-rank (Mantel–Cox) test (**P < 0.01; ***P < 0.001). **f** Bar graph depicting semi-quantitative analysis of islet area in pancreatic sections obtained from ecto-TMEM219-treated and untreated NOD mice after 14 weeks of treatment (*n* = 4/group). Data are expressed as a score ranging between 0 and 4. **g** Bar graph showing reduction of peripheral IGFBP3 levels in ecto-TMEM219-treated and untreated NOD mice after 14 weeks of treatment (*n* = 5). **h** Bar graph depicting peripheral insulin levels measured in ecto-TMEM219-treated (*n* = 4 and 7, respectively) and untreated NOD mice after 14 weeks of treatment (*n* = 4). **i** Bar graph showing peripheral IGF-I levels in ecto-TMEM219-treated and untreated NOD mice after 14 weeks of treatment (*n* = 5). **j** Bar graph representing islet infiltration detected in pancreatic sections obtained from ecto-TMEM219-treated as compared to untreated NOD mice after 14 weeks of treatment (*n* = 3/group). Data are expressed as a score ranging between 0 and 4. **k** Representative H&E staining in serial pancreatic islet tissue sections obtained from 24-week-old NOD mice treated with prolonged ecto-TMEM219 or from untreated mice shown as control (*n* = 3/group). ×10 original magnification, scale bar, 300 μm. **l** Bar graph showing IFN-γ-producing cells upon in vitro re-stimulation with BDC2.5 or IGRP peptides detected by ELISpot in ecto-TMEM219-treated as compared to untreated NOD mice after 14 weeks of treatment (*n* = 10/group, *n* = 6 in the short course ecto-TMEM219-treated group). **m**, **n**, **o** Bar graphs representing the percentage of CD4^+^CD44^hi^CD62L^lo^ (*n* = 4/group), CD8^+^CD44^hi^CD62L^lo^ (*n* = 4/group), and CD4^+^CD25^+^Foxp3^+^ cells (*n* = 3/group) detected in splenocytes of ecto-TMEM219-treated as compared to untreated NOD mice after 14 weeks of treatment. **p**, **q** Newly hyperglycemic NOD mice were treated with Ecto-TMEM219 or were left untreated and the incidence of diabetes was then compared using the log-rank (Mantel–Cox) test (***P < 0.001). **r** Representative H&E staining in serial pancreatic islet tissue sections obtained from newly hyperglycemic NOD mice, treated with ecto-TMEM219 or from untreated mice shown as control (*n* = 3/group). ×20 original magnification, scale bar, 100 μm. Data are expressed as mean ± standard error of the mean (SEM) unless otherwise reported. *P < 0.05; **P < 0.01; ***P < 0.001; ****P < 0.0001 by one-way ANOVA followed by Bonferroni’s post hoc test, log-rank Mantel–Cox test, Kruskal–Wallis test adjusted for multiple comparisons. mRNA expression was normalized to *Gapdh*. The data shown in (**b**), k1–k2, r1–r2 are the representative result from three independent experiments. Source data are provided as a Source Data file. NOD nonobese diabetic mice, LtNglt long-term normoglycemic NOD mice, NOR nonobese diabetes-resistant mice, hyper hyperglycemic, mAb monoclonal antibody, Arb. units arbitrary units.

We finally investigated the role of the IGFBP3/TMEM219 axis and its targeting in the NOD mouse, which spontaneously develops T1D and recapitulates in vivo its features. First, we demonstrated that TMEM219 is expressed in pancreatic islets of NOD mice (Fig. 5a, b). Next, we paralleled our observations in human patients by demonstrating elevated peripheral IGFBP3 levels in pre-diabetic and diabetic non-obese-diabetic (NOD) mice (Fig. 5c). In particular, higher IGFBP3 plasma levels were found in 4-weeks and 10-week-old NOD mice, which are not hyperglycemic yet and in which beta cells are still detectable but already exhibit insulitis and islet-autoimmunity. However, in long-term normoglycemic NOD mice, which represent ~20–30% of female NOD mice that are normoglycemic at 26–30 weeks of age and do not develop diabetes, and in NOR mice resistant to diabetes, peripheral IGFBP3 levels were not increased (Fig. 5c). As observed in human subjects, peripheral IGF-I levels were not altered in pre-diabetic and diabetic NOD mice (Fig. 5d), resulting in an elevated IGFBP3/IGF-I ratio (Supplementary Fig. 6j), which again highlighted the availability of a peripheral IGFBP3 fraction that may bind TMEM219 on islet cells. To better understand the role of IGFBP3/TMEM219 in controlling the beta-cell mass in vivo in disease conditions such as T1D, we pharmacologically modulated IGFBP3-mediated signaling with ecto-TMEM219 in a T1D prevention study in 10-week-old normoglycemic NOD mice. IGFBP3/TMEM219 blockade with ecto-TMEM219 prevented the onset of diabetes in nearly 75% of treated mice (up to 80%), while only 25% of control mice were protected from diabetes in the untreated group (Fig. 5e). Islet pathology studies demonstrated significant preservation of islet morphology and area in ecto-TMEM219-treated mice, with low islet infiltration and reduced peripheral IGFBP3 levels as compared to untreated controls (Fig. 5f–j). Of note, prolonged IGFBP3/TMEM219 blockade with ecto-TMEM219 was associated with significant beta-cell expansion, which lead to a significant increase of both islet area and peripheral insulin levels (Fig. 5f, h, k and Supplementary Fig. 6k), mimicking nesidioblastosis. Mechanistic studies demonstrated a reduction in the autoimmune response in ecto-TMEM219-treated NOD mice, when splenocytes were re-challenged in vitro with the CD4 and CD8-restricted islet peptides BDC2.5 and IGRP in an IFN-γ ELISpot assay (Fig. 5l). This observation was paralleled with a slight decrease in effector memory CD8 T cells and a small increase in Treg population observed by flow cytometric analysis (Fig. 5m–o). Finally, ecto-TMEM219 was tested in a diabetes reversal NOD mouse model, in which hyperglycemic NOD mice were treated with ecto-TMEM219 within 24 h from the onset of hyperglycemia and demonstrated preserved blood glucose levels, with 25% of mice remaining normoglycemic until the completion of the study at day 20 (Fig. 5p, q), and no detectable islet infiltration/inflammation (Fig. 5r). In summary, these data demonstrate a protective effect of targeting the IGFBP3/TMEM219 axis on beta cells in vivo also in autoimmune diabetes.

## Discussion

While factors that promote beta-cell growth and replication have been extensively studied^17,23,24^, the mechanisms that lead to beta-cell loss remain far from being fully understood. Several stress factors have been identified as playing a role in beta-cell dysfunction and injury^3,4^. The present study demonstrates that the death receptor TMEM219 is expressed in islet beta cells and that, when bound to its ligand IGFBP3, TMEM219 induces Caspase-8-dependent apoptosis of beta cells, which leads to beta-cell loss. Moreover, the addition of IGFBP3 in vitro alters the insulin secretory machinery in beta cells, resulting in beta-cell dysfunction. The observation that IGFBP3 levels are increased in patients with T1D or T2D and in those at risk for developing diabetes, as well as in pre-diabetic and diabetic mice, suggests a dysregulation of the IGFBP3/TMEM219 pathway in the context of diabetes. Conflicting and too little data exist in the literature with regard to IGFBP3 circulating levels in patients with T1D^25^ and T2D^26,27^, which are often assessed in heterogeneous populations of patients (Supplementary Table 7), thus requiring further in-depth studies. Also, proteolysis of IGFBP3 have been detected at a high level in serum of patients with T1D^28,29^ and confirmed in our immunoprecipitation studies. Indeed, in preclinical models, constitutive overexpression of IGFBP3 was associated with reduced islet mass and dysglycemia^21^, and our results demonstrate that short-term IGFBP3 exposure in vivo alters glycometabolic control in naive mice, although diabetes itself may alter IGFBP3 distribution and clearance^30^, thus indicating that elevated IGFBP3 levels are involved in beta-cell loss/dysfunction. As a proof-of-concept, blockade of IGFBP3/TMEM219 signaling in vitro by silencing TMEM219 via siRNA delivery prevented IGFBP3-mediated apoptosis/damage of beta cells and restored insulin expression and secretion. More importantly, the use of a newly generated recombinant protein based on the TMEM219 extracellular domain (ecto-TMEM219), which inhibits the IGFBP3/TMEM219 pathway, protected beta cells in vitro and prevented the onset of hyperglycemia in murine models of diabetes in vivo. This was also confirmed when TMEM219 was genetically deleted on beta cells. Interestingly, prolonged inhibition of TMEM219 signaling using ecto-TMEM219 treatment appeared to prevent normal beta-cell turnover and increased peripheral insulin levels to the point that the beta-cell mass markedly expanded, almost mimicking nesidioblastosis. We should also acknowledge a possible effect on the immune system. The limited IGFBP3-mediated beta-cell loss may result in a reduced delivery of islet autoantigens to the circulation and to pancreatic lymph nodes, thus dampening the priming/activation of autoreactive T cells. Overall, our findings demonstrate that the IGFBP3/TMEM219 pathway may serve as a physiological controller of beta cells and their lifespan, and that this pathway is dysregulated in diabetes. As reduced beta-cell mass and function are commonly observed in T1D and T2D^2,31,32^, usually associated with increased beta-cell apoptotic rate^12,14,33–35^, discovery of surface beta-cell receptors that negatively regulate cell survival and that may make beta cells more susceptible to stress factors or to immune-mediated attack is of significant importance in order to understand ways in which the beta-cell pool can be preserved. The increased hepatic production of IGFBP3 observed in vitro upon exposure to either T1D or T2D serum and the abrogation obtained by targeting the pro-inflammatory microenvironment further suggest a common path of dysregulation for the IGFBP3/TMEM219 axis in diabetes. We cannot exclude that the use of ecto-TMEM219 may interfere with other factors such as IGF-I, IGF-II, and other IGFBPs (e.g., IGFBP1), including their interaction with the IGF-IR, all potentially involved in regulating beta-cell homeostasis, although our data do not suggest the existence of the aforementioned issues. In summary, when abnormally released into the circulation, IGFBP3 may act as a circulating toxic factor for beta cells (*Betatoxin*) that ultimately causes beta-cell death and dysfunction through TMEM219 activation. The discovery of circulating factors involved in the initiation/facilitation of beta-cell injury and associated with a reduction in beta-cell mass in diabetes is of significant importance for the development of therapies for T1D and T2D. Such discoveries may pave the way for therapeutic approaches capable of halting or delaying the disease by targeting the process that ultimately leads to insulin deficiency. Blockade of IGFBP3/TMEM219 may thus represent a novel therapeutic option for diabetic patients to preserve their insulin-producing beta-cell pool.

## Methods

A detailed description of the methods used in this study is provided in the Supplementary Methods section. Our research complies with all relevant ethical regulations of the University of Milan.

### Patients and study design

#### T1D study

Plasma samples from 30 patients with type 1 (T1D), from 30 patients screened positive for islet autoantibodies (AutoAb^+^), from 45 patients with new-onset T1D (10 days, new-onset T1D) and from 30 healthy volunteers (CTRL) were obtained with informed consent as reported in Supplementary Table 1. T1D patients were undergoing intensive insulin treatment at the time of enrollment in the study, while the CTRL, the AutoAb^+^ and the new-onset T1D group were not being administered any medication. All T1D subjects were on the same treatment with regard to antiplatelet therapy (ASA) and anti-hypertension (angiotensin-converting enzyme inhibitors). This study was conducted in accordance with Institutional Review Board approvals (ImmunoT1D, NPOD) and registered as NCT03794739.

#### T2D study

Plasma samples from normal glucose tolerant (*n*: 235; NGT), impaired glucose tolerant (*n*: 200; IGT), and newly diagnosed type 2 diabetes (*n*: 81; T2D) individuals were collected at the University of Pisa (Italy) under the Genfiev protocol study. The study enrolled patients at risk for developing T2D, including those with NGT, IGT, and IFG but excluding those with T2D. NGT, IGT, and T2D classifications were determined based on the results of OGTT tests according to the ADA 2003 criteria^36^. Analysis of IGFBP3 levels was successfully performed and determined for each group as follows: IGT: *n* = 149/200; NGT: *n* = 146/235, and newly diagnosed T2D: *n* = 70/81. Demographic and main clinical characteristics are reported in Supplementary Table 2. Concomitant treatment, inclusion, and exclusion criteria are described and reported at the website https://clinicaltrials.gov/ct2/show/record/NCT00879801?term=GENFIEV.

#### Liver transplantation study

Samples were also obtained from 20 liver-transplanted patients with diabetes before and after transplantation at 2 years of follow-up^37^. Ten out of 20 patients remained diabetic at the time of the 2-year follow-up (non-regressor), while ten patients near-normalized their glycometabolic control (regressor). Demographic and clinical characteristics are reported in Supplementary Table 3. Inclusion and exclusion criteria of patients enrolled in the study have been registered under the NCT02038517.

#### Islet transplantation study

Plasma samples were also obtained from 17 islet-transplanted patients with T1D before and after transplantation with a mean ± SEM follow-up of 16.3 ± 1.6 months. Baseline characteristics were as follows (mean ± SEM): HbA1c: 7.3 ± 0.2% (56.5 ± 2.4 mmol/mol); age: 54.5 ± 2.4 years; history of T1D: 41.0 ± 2.7 years. With regard to immunosuppressive treatment, 13 patients were on mycophenolate mofetil plus tacrolimus, 2 patients were on mycophenolic acid delayed release plus tacrolimus, 1 patient was on tacrolimus alone and 1 patient received alemtuzumab with mycophenolate mofetil plus tacrolimus. A measurement of C-peptide <0.4 ng/ml was considered indicative of failing islet transplantation.

#### Obese T2D study

Serum samples were finally obtained from 18 obese diabetic patients selected for bariatric surgery treatment whose baseline characteristics were as follows (mean ± SEM): HbA1c: 8.0 ± 0.3% (63.5 ± 3.6 mmol/mol); fasting plasma glucose: 167.5 ± 12.1 mg/dl; age: 49.3 ± 2.6 years; BMI: 40.0 ± 1.5 Kg/m^2^.

Samples were also obtained from the Network for Pancreatic Organ donors with Diabetes (nPOD) program within the project entitled “Role of TMEM219 Expression in Type 1 Diabetes”. An approval for the use postmortem tissues was already included in the nPOD protocol (Local Medical Ethical and Institutional Review Board (Milan, area 1). All subjects provided informed consent prior to study enrollment. Studies not included in the routine clinical follow-up were covered by an appropriate Institutional Review Board approval at each Institution (Supplementary Table 4). All studies were conducted in compliance with all relevant ethical regulations for studies involving human subjects.

### Pancreatic islets

Human islets were isolated from 11 cadaveric organ donors with the following mean ± SEM: age: 49.3 ± 1.4 years, and purity: 74 ± 7%, according to the procedure previously described^38^ in conformity with the ethical requirements approved by the Niguarda Cà Granda Ethics Board, which included an approval for non-viable human islets preparations to be used for research purposes. Briefly, islets were isolated using the automated method previously described^39–41^. Two types of enzymes were used: collagenase type P (1–3 mg/ml) and liberase (0.5–1.4 mg/ml) (Roche, Indianapolis, IN). Islets were purified by the discontinuous gradient in syringes (density gradient: 1,108; 1,096; 1,037: EuroFicoll, (Sigma-Aldrich, Milan, Italy), or by continuous gradient with refrigerated COBE processor as previously described^42^. After isolation, islets were cultured at 22 °C in a humidified atmosphere (5% CO_2_), in CMRL (Mediatech, Cellgro, Manassas, VA) supplemented with 10% FBS, 100 U/ml penicillin, 100 μg/ml streptomycin sulfate (Euroclone, Celbio), and 2 mmol/l glutamine (Mediatech, Cellgro). In vitro characterization and culture of islets was performed on islet material processed within 72 h after isolation. Human pancreatic islets of Langerhans (#35002-04) isolated from healthy subjects were also purchased from a commercial source (Celprogen, Torrance, CA) and cultured with standard medium and 10% FBS as per the manufacturer’s instructions and as previously described^43^. Murine islets were kindly provided by Prof. James Markmann (Transplantation Unit, Department of Surgery, Massachusetts General Hospital, Harvard Medical School, Boston, MA)^44^. Pancreatic islets were isolated from 5 to 10 8-week-old C57BL/6J (B6) mice by collagenase digestion followed by density gradient separation and then hand-picking, as described previously^19,45^. Islets were then plated and cultured in RPMI 1640 medium supplemented with l-glutamine, penicillin and 10% with a glucose concentration of 5 mM for 72 h.

### Pathology and immunohistochemistry

Hematoxylin & eosin (H&E) staining for morphological evaluation and immunohistochemistry expression studies were conducted on murine tissue samples as previously described^46^. The following antibodies were used: insulin (A0564, DAKO, CiteAb, Bath, UK) and anti-TMEM219 primary antibody (polyclonal, 1:100, Sigma HPA059185). These antibodies were immunohistochemically tested in pancreatic tissues of human subjects, Beta-TMEM219^−/−^/wild-type and NOD mice.

### Immunofluorescence

Immunofluorescence samples were examined using a confocal system (TCS SP2 Laser Scanning Confocal, Leica, Wetzlar, Germany). Images were acquired in multitrack mode, using consecutive and independent optical pathways. The following primary antibodies were used for staining of human tissues/cells: rabbit polyclonal TMEM219 (1:100, Sigma, HPA059185), guinea pig polyclonal insulin (1:200, DAKO, A0564). The following primary antibodies were used for staining of murine tissues/cells: rabbit polyclonal TMEM219 (1:100 or 1:50, Sigma, HPA059185), guinea pig polyclonal insulin (1:200, DAKO, A0564), mouse monoclonal IGFBP3 antibody (1:100, LSBio, C45037). The following secondary antibodies were used for staining of human or murine tissues/cells: donkey anti-rabbit FITC (1:200), donkey anti-mouse FITC (1:200), donkey anti-guinea pig TRITC (1:200), and donkey anti-rabbit TRITC (1:200), donkey anti-mouse 550 (1:200), (all from Jackson ImmunoResearch, West Grove, PA). ApopTag Plus Peroxidase In Situ Apoptosis Detection kit (Millipore, Billerica, MA) was used for apoptosis detection^47^, and staining was performed using sheep polyclonal antibody (FITC). Human and murine pancreatic islets co-cultured with/without IGFBP3 (R&D Systems, 8874-B3, Minneapolis, MN, 50 ng/ml), with/without ecto-TMEM219 (generated by us in collaboration with GenScript, Piscataway, NJ, 130 ng/ml, 1:1 ratio with IGFBP3), were stained for TMEM219, insulin and with Apoptag for colocalization studies by immunofluorescence. Murine and human beta cells were co-cultured in the same conditions and stained similarly to pancreatic islets. Islets and beta cells were fixed in 10% neutral buffered for 30 min, washed with PBS three times and permeabilized with PBS containing 2% BSA and 0.3% Triton X-100 for 20 min, blocked with 10% serum, and finally incubated with primary antibodies overnight at 4 °C and subsequently labeled with fluorescent secondary antibodies for 2 h at room temperature. Primary and secondary antibodies are described above.

### Islet and beta-cell in vitro studies and characterization

#### Culturing conditions

Human and murine islets and human beta-cell lines were cultured with/without IGFBP3 (R&D Systems, 8874-B3, 5, 50, 500 ng/ml), with/without ecto-TMEM219 (65, 130, 260 ng/ml in a 1:2, 1.1, and 2:1 molar ratio) as detailed in “Recombinant proteins and interventional studies“, and then were used for immunofluorescence studies, RNA extraction, apoptosis detection, and protein analysis. Supernatants were collected to measure insulin release and IGFBP3 by ELISA. To mimic diabetic conditions, human diabetic serum obtained from established T1D or T2D patients (*n* = 5/group) was pooled and added in place of regular FBS at a concentration of 10% to human islets/beta-cell lines, which were cultured as reported in “Pancreatic islets and Beta cell line” and compared with the addition of pooled CTRL serum (*n* = 5) at a concentration of 10% (in place of regular FBS) or regular culturing medium (with 10% FBS). After 72 h, islets/cells were collected, and apoptosis/cell death as well as expression of *INS* and *CASP8* (Life Technologies) were examined using ELISA (Roche Diagnostics GmbH, 11544675001, Mannheim, Germany), immunofluorescence, and RT-PCR.

#### Transcriptome profiling

Total RNA was isolated from purified islets/beta-cell lines using the RNeasy Mini Kit (Qiagen, Valencia, CA) with on-column DNase I digestion. Next, 3 µg total RNA from each sample was reverse-transcribed using the RT2 First Strand kit (C-03; SABiosciences, Frederick, MD). The Profiler PCR Arrays quantitatively measure the expression of a panel of genes using SYBR Green-based real-time PCR. To assess the transcriptome profile of apoptotic markers and beta-cell-related markers, Human Apoptosis PCR Arrays (PAHS-012Z, SABiosciences) and Human Insulin Signaling Pathway PCR Arrays (PAHS-030Z, SABiosciences) were used (Supplementary Data 1). With regard to TMEM219 and CASP8 mRNA expression, data were obtained from an Affymetrix Human Gene 2.0 ST array analysis previously performed on laser-captured islets of non-diabetic subjects, T1D, T2D patients, and AutoAb^+^ patients provided by Prof. Ivan Gerling^48^. Data were expressed as fold change as compared to gene expression of healthy subject samples.

#### RNAseq analysis

RNA was extracted from at least 400 purified islets of non-diabetic donors (*n* = 4, 3 males and 1 female, mean ± SEM: age: 50.7 ± 1.8 years, and purity: 82 ± 4%) whose pancreata were not suitable for donation processed by the Niguarda Hospital, using the Direct-zol RNA Mini prep Plus kit (R2070, Zymo Research, Irvine, CA) and RNAseq was performed with RNA quality assessed using an Agilent RNA Nano Chip, and run on Bioanalyzer 2100 (Agilent, Santa Clara, CA) (RNA sequencing was performed by the Center of Bioinformatics and Functional Genomics at OSR). Briefly, library preparation was performed using the Illumina TruSeq Stranded mRNA kit (Illumina, San Diego, CA), with 300 ng of total RNA as per the manufacturer’s instructions. Sequencing was performed according to the TruSeq SR protocol (Illumina) using NextSeq 500 High 75 Sequencing System (Illumina), yielding an average of 40 × 10^6^ reads per sample. Gene expression analysis was performed using R version 3.6.1 (libraries: edgeR_3.26.5, DESeq2_1.24.0, pheatmap_1.0.12), and transcripts were assembled and normalized to reads per kilobase per million mapped reads (RPKM) expression units to estimate the relative abundance of transcripts. Data obtained were further subjected to a rank analysis, with regard to those genes previously identified by transcriptome analysis (Affymetrix), as surface proteins/receptors expressed to a moderate/high level (cutoff >25) in human islets and beta cells^49^. Based on their RPKM, selected genes were then grouped according to a cutoff of 0–25, 25–50, 50–75, and 75–100. Genes with RPKM falling within the 75–100 cutoff were considered highly expressed (Supplementary Data 2).

#### qRT-PCR analysis

RNA from purified human/murine islets/beta-cell line was extracted using TRIzol Reagent (Invitrogen, Carlsbad, CA), reverse-transcribed using Super Script II Reverse Transcriptase (Invitrogen) and qRT-PCR analysis was performed using TaqMan assays (Life Technologies) according to the manufacturer’s instructions. Normalized expression values were determined using the ΔCt method. Quantitative reverse transcriptase polymerase chain reaction (qRT-PCR) data were normalized for the expression of ACTB, and ∆∆Ct (fold change) or ∆Ct values were calculated. Statistical analysis compared gene expression across all cell populations for each patient via one-way ANOVA followed by a Bonferroni post-test for multiple comparisons between the population of interest and all other populations. Statistical analysis was performed also using the software RT^2^ profiler PCR Array Data Analysis (Qiagen). For comparison between two groups, a Student’s *t* test was employed. Analysis was performed in triplicates before/after 72 h of culture. Reported on Supplementary Table 8 are the characteristics of primers used for human and murine genes.

#### ELISA

IGFBP3 and IGF-I levels in the individual or pooled sera/plasma of all groups of subjects and in all groups of treated and untreated mice were assessed using commercially available ELISA kits, according to the manufacturer’s instructions (Sigma Aldrich/Millipore RAB0235 and R&D Systems (Minneapolis, MN) SG100; R&D Systems MGB300 and SMG100). Huh7 were cultured for 72 h in regular medium and were exposed to different culturing conditions (please see above section, “Recombinant proteins and interventional studies”). Culturing supernatant was collected, and IGFBP3 levels were assessed using an IGFBP3 ELISA kit (Sigma Aldrich, RAB0235) according to the manufacturer’s instructions. Collected cells were dissociated with trypsin and counted using a hemacytometer. Insulin levels were assayed in serum/plasma and culturing supernatant with a microparticle enzyme immunoassay (Mercodia Iso-Insulin ELISA, 10-1113-01 and 10-1247-01, Uppsala, Sweden) with intra- and inter-assay coefficients of variation (CVs) of 3.0% and 5.0%. TMEM219 expression was quantified in lysates of human islets and human beta-cell lines using an ELISA kit (MyBioSource ELISA, MBS9341285, San Diego, CA) according to the manufacturer’s instructions. Human podocytes (Podo, kindly provided by Dr. M.A. Saleem), hepatocytes-derived cell line (Huh7, kindly provided by Division of Genetics and Cel Biology San Raffaele Scientific Institute) were used as negative controls, a breast cancer cell line (tMCF7, maintained in Fiorina’s lab) stably transfected with TMEM219 (TMEM219 GFP-tagged, Origene, Rockville, MD, RG207974) was used as a positive control. Murine TMEM219 expression was quantified in lysates of the whole pancreas and collagenase-dissociated murine islets by using an ELISA kit (MyBioSource ELISA, MBS9335105) according to the manufacturer’s instructions.

#### Luminex analysis

IGFBP3, IGF-I, and IGF-II plasma levels were also measured by using Luminex kit HIGFBMAG-53K (MILLIPLEX MAP Human IGF Binding Protein Magnetic Bead Panel) and HIGFMAG-52K (MILLIPLEX MAP Human IGF-I, II Magnetic Bead Panel) from Merck Millipore (Burlington, MA).

#### Electrochemiluminescent Immunoassay

IGFBP3 measurement was validated by using a clinically grade electrochemiluminescence immunoassay (IGFBP3 ELECSYS E2G 100 # 07574720190, Roche Diagnostics S.p.A, Switzerland.).

#### Apoptosis/cell death analysis

To assess apoptosis/cell death in purified human islets and in beta-cell lines, we employed a photometric enzyme immunoassay (Roche Diagnostics GmbH, 11544675001, Mannheim, Germany), which quantifies in vitro the histone-associated DNA fragments after induced cell stress on cell cytoplasmic lysates and cell supernatants.

#### Islets functional studies

Following the guidelines of the NIH Clinical Islet Transplantation Consortium for islet functional studies^50^, after overnight culture at 37 °C (24 h), purified human islets were incubated with media containing a low glucose concentration (2.8 mM) and cultured with/without IGFBP3 and with/without ecto-TMEM219. Then the same islets were incubated with media containing a higher glucose concentration (28 mM) for 2 h. A sample of the supernatant was collected during low and high-glucose conditions. The amount of insulin present in both the supernatant samples was measured using a commercially available Human Insulin ELISA kit (Mercodia Iso-Insulin ELISA, 10-1113-01, Uppsala, Sweden). The stimulation index was calculated by dividing the insulin concentration of the supernatant from the 28 mM glucose incubation by the insulin concentration of the supernatant from the 2.8 mM glucose incubation^50^.

#### Cleaved Caspase 8 and IGFBP3/TMEM219 signaling analysis

Human pancreatic islets cultured with/without IGFBP3, with/without ecto-TMEM219, and in the presence/absence of TMEM219 siRNA were lysed and processed for ELISA (MyBiosource ELISA, MBS766157) to quantify Cleaved Caspase 8. Akt and phosphorylated-AKT, ERK1/2 and phosphorylated-ERK1/2 were detected by using the following antibodies (all from Cell Signaling Technology, Danvers, MA, USA): Phospho-AKT (Ser473, D9E) XP^®^ mAb (#4060) and panAKT (C67E7) mAb (#4691), Phospho-p44/42 MAPK mAb (Erk1/2, Thr202/Tyr204, #9101 S) and p44/42 MAPK mAb (Erk1/2, #9102 S), all used at 1:1000 concentration in TBS-T + 5% nonfat dry milk. Goat anti-Rabbit IgG (whole molecule) – Peroxidase antibody (1:5000, Merck, A0545) was used as a secondary antibody. The immunoblotting procedure is described in Supplementary Methods. Briefly, after incubation with appropriate primary and secondary antibodies, signals were revealed using enhanced chemiluminescence (Thermo Fisher Scientific) and visualized by a Uvitec Chemidoc mini hd9 imaging system (Uvitec, Cambridge, UK). Densitometric analysis was performed using Uvitec in-house software and expressed as mean ± SEM of data from at least three independent experiments.

#### Flow cytometry

Human pancreatic islets of non-diabetic subjects were cultured with standard medium, or in the presence/absence of IGFBP3 and/or in the presence/absence of Ecto-TMEM219 for 72 h as previously described (see “Pancreatic islets and Interventional studies”). Islets were detached with Versene (ThermoFisher Scientific) and stained with the BD Horizon™ Fixable Viability Stain 510 (FVS510, BD Biosciences 564406, San Jose, CA), which ensures accurate assessment of cell viability in samples after fixation and/or permeabilization as per manufacturer’s instructions. After being stained with BD Horizon™ Fixable Viability Stain 510, cells were next fixed and permeabilized with Fixation and Permeabilization Solution Kit (554714, BD Biosciences, San Jose, CA)^51^ and finally stained with guinea pig anti-insulin antibody (1:200, Guinea Pig, ThermoFisher Scientific, PA1-26938) followed by AlexaFluor488 anti-guinea pig (1:200, ThermoFisher Scientific, A-11073). Flow cytometry analysis was performed using a BD FACS Celesta flow cytometry system (BD Biosciences) and analyzed using Flowjo software (Version 6 and Version 10, Tree Star, Ashland, OR).

#### Flow sorting

Human pancreatic islets of non-diabetic subjects were cultured with standard medium, or in the presence of IGFBP3 for 72 h as previously described (see “Pancreatic islets and Interventional studies”), and dissociated by trypsinization. In order to select insulin-positive and -negative populations, the cell fraction obtained was permeabilized using the Fixation and Permeabilization Solution Kit (554714, BD Biosciences) and stained with an APC-conjugated anti-insulin antibody (1:100, IC1417A, R&D Systems). Stained cells were flow-sorted using MoFlo Legacy (Beckman Coulter, Brea, CA) and analyzed by qRT-PCR.

### Immunoprecipitation

Beta cells (Blox-5) were cultured with pooled T1D serum (*n* = 5 donors) for 16 h as described in culturing conditions and then treated with a cross-linker reagent (DTSSP, Thermo Scientific) according to the manufacturer’s protocol. Cells were then incubated on ice for 45 min in NP-40 Cell Lysis Buffer (50 mM Tris, pH 7.4 250 mM NaCl, 5 mM EDTA, 50 mM NaF, 1 mM Na3VO4, 1% NonidetTM P40 (NP40), 0.02% NaN3), and protease inhibitor cocktail (Sigma-Aldrich). The total protein content of cell lysates was measured with the Bradford assay. Dynabeads Protein G (Life Technologies) were used for immunoprecipitation according to the manufacturer’s instructions. Dynabeads were pre-incubated with polyclonal rabbit anti-TMEM219 (10 µg, Sigma-Aldrich) antibody before the addition of lysates. Precipitates were then evaluated by immunoblotting with mouse anti-IGFBP3 (LS-C340284, LSbio) diluted 1:1000 followed by anti-mouse HRP (A9044 Sigma-Aldrich, diluted 1:2500) and the resulting bands were visualized using a Uvitec Chemidoc mini hd9 imaging system (Uvitec, Cambridge, UK).

### Recombinant proteins and interventional studies

In total, 50 ng/ml recombinant human IGFBP3 (R&D Systems, 8874-B3) and 130 ng/ml ecto-TMEM219 (Genscript, 1:1 molar ratio vs. IGFBP3)^52^ were added to islets/cell cultures at day +1 from islets collection/cell culture. Those concentrations were selected upon a dose-titration in vitro validation (Supplementary Fig. 4a, b). Recombinant human IGFBP3 was expressed in S9-baculovirus, with a molecular weight of 30 kDa and a purity >95%^53^. Ecto-TMEM219 protein, cloned on the TMEM219 extracellular domain and consisting of the following 162 amino acids (THRTGLRSPDIPQDWVSFLRSFGQLTLCPRNGTVTGKWRGSHVVGLLTTLNFGDGPDRNKTRTFQATVLGSQMGLKGSSAGQLVLITARVTTERTAGTCLYFSAVPGILPSSQPPISCSEEGAGNATLSPRMGEECVSVWSHEGLVLTKLLTSEELALCGSR) was obtained by Genscript from inclusion bodies, one-step purification through Nickel column by using *E. coli* expression Vector pET30a, in a buffer containing 10 mM HEPES, 0.05% SDS with a pH 7.5. Expression purity was >90% as per SDS indication and the molecular weight was 18 kDa. Specificity of ecto-TMEM219 in binding IGFBP3 has been demonstrated in vitro in competitive binding assays and affinity between IGFBP3 and ecto-TMEM219 has been validated by Biacore 8 K with a KD of 1.01E-06 M (Supplementary Fig. 7a–g). A putative 3D representation of ecto-TMEM219 obtained through bioinformatic analysis is reported in Supplementary Fig. 7h. The neutralizing anti-IGFBP3 monoclonal antibody was obtained by Evitria through phage display-based generation (Yumab GmbH, Braunschweig, Germany) and added in vitro at a concentration of 10 µg/ml (50:1 molar ratio vs. IGFBP3). Anti-IGFBP3 monoclonal antibody was a generous gift from Enthera s.r.l. Recombinant human IGF-I (100 ng/ml, Sigma, I3769), and anti-IGF-IR (1 μM, Selleckchem, Boston, OSI-906) were added to cell cultures at day +1 from islets collection/cell culture. The human immortalized hepatocytes-derived cell line HuH7 was cultured in DMEM with 10% FBS and it was exposed to high-glucose concentration (35 mM), to pro-diabetogenic conditions (a mixture of 2 ng/ml recombinant human IL-1β (R&D Systems, 201-LB-005), and 1000 U/ml recombinant human IFN-γ (PeproTech, 300-02)), with/without anti-IL-1β (10 µg/ml, ThermoFisher Scientific, MA5-23691)/anti-IFN-γ (10 µg/ml, ThermoFisher Scientific, 16-7318-81), or to T1D/T2D/CTRL serum, which was added to the culturing medium in place of regular FBS (10%), for 72 h. Ecto-TMEM219 was administered in vivo to 10-week-old NOD mice and to C57BL/6J mice fed a high-fat diet (HFD-B6) mice intraperitoneally (i.p.) at a dose of 0.1 mg/mouse/day for 10 days (short-term) and 0.1 mg/mouse/day for 10 days and twice per week for 10 weeks (long-term) for NOD mice, and 0.1 mg/mouse every other day for 5 weeks for HFD-B6 mice. Ecto-TMEM219 was also administered to NOD mice who developed blood glucose levels >250 mg/dl for 24 h at the dose of 0.1 mg/mouse/day for 10 days in a diabetes reversal study. Recombinant IGFBP3 was administered in vivo to 8-week-old B6 mice i.p. at a dose of 0.2 mg/mouse/day for 5 days. To create beta-cell-specific TMEM219^-/-^ mice, mice harboring exon 4 of the TMEM219 gene flanked by loxP sites (TMEM219^flox/flox^) were crossed with mice expressing Cre under the insulin-1 gene initiation codon (B6(Cg)-Ins1tm1.1(cre)Thor/J) from the Jackson Laboratories (Bar Harbor, ME). Mice were injected with tamoxifen (20 mg/ml, 100 μl; T5648 Sigma-Aldrich) at days 0 and +1 in order to activate the Cre-recombinase deletion of the floxed sequences.

### Small RNA interference

Purified human islets obtained from non-diabetic donors, or cells from a human beta-cell line, were grown in appropriate medium, or in some experiments in culturing medium modified by adding CTRL/T1D/T2D serum in place of regular FBS as previously described (please see “Islets and beta-cell in vitro studies and characterization”). After 72 h of culture, which allowed the islets cells from the beta-cell line to recover, 750 ng of small interfering RNA (siRNA; Flexitube siRNA SI04381013, Qiagen) in 100 μl culture medium without serum and with 6 μl HiPerFect Transfection Reagent (Qiagen) were incubated at room temperature to allow for the formation of transfection complexes. Islets or cells from the beta-cell line were incubated with these transfection complexes under their normal growth conditions for 6 h. Analysis of gene silencing was performed at 24, 48, and 72 h by evaluating mRNA of *CASP8* and *INS* expression, cleaved Caspase 8, phosphorylated AKT, total AKT, total ERK1/2, and phosphorylated ERK1/2. Control siRNA was used as a negative control.

### Animal studies

C57BL/6J (B6) male mice, female non-obese diabetic (NOD/ShiLtJ) mice, female non-obese diabetes-resistant (NOR/LtJ), and INS1^cre^ (B6(Cg)-Ins1^tm1.1(cre)Thor^/J) were obtained from the Jackson Laboratories. TMEM219^flox/flox^ were generated in collaboration with Applied StemCell (Milpitas, CA) and housed at Charles River Laboratories (Wilmington, MA). TMEM219^flox/flox^INS1^cre^ male mice (Beta-TMEM219^−/−^) were obtained by breeding TMEM219^flox/flox^ mice with INS1^cre^ mice, and the resulting colony was housed at Charles River Laboratories. Mice were housed at a temperature between 21 and 23 °C with 50–60% humidity and kept on a 12/12 h light/dark cycle with free access to food and water. All mice were cared for and used in accordance with institutional guidelines approved by the Boston Children’s Hospital Institutional Animal Care and Use Committee or in accordance with Italian law on animal care N° 116/1992 and the European Communities Council Directive EEC/609/86. All animal studies were approved by the Italian Ministry of Health and Local University of Milan Committee and by the Boston Children’s Hospital Institutional Animal Care and Use Committee. Some studies were conducted at the Jackson Laboratories.

#### Diabetes monitoring

Overt diabetes was defined as blood glucose levels above 250 mg/dL for 3 consecutive days. Blood glucose was measured using the Breeze2 (Bayer S.p.A) blood glucose meter.

### Statistical analysis

Continuous variables are presented as means with standard errors, and categorical variables are presented as proportions. We used independent sample *t* tests to compare continuous variables and chi-square test/Fisher’s exact test to compare categorical variables. For multiple comparisons, one-way or two-way ANOVA followed by Bonferroni/Holm–Sidak post hoc test between the group of interest and all other groups were used. Diabetes incidence among different groups was analyzed with the log-rank (Mantel–Cox) test. Two-tailed *P* values of less than 0.05 were considered statistically significant. All the analyses were performed by using GraphPad Prism V7 or Microsoft Excel V16.43.

### Reporting summary

Further information on research design is available in the Nature Research Reporting Summary linked to this article.

## Supplementary information

Supplementary Information

Description of Additional Supplementary Files

Supplementary Data 1

Supplementary Data 2

Reporting Summary

## Data Availability

The data supporting the findings from this study are available within the article file and its Supplementary Information. The raw sequence data and partially processed data have been deposited in Genome Sequence Archive for Human “PRJNA782561”. Any other raw data or non-commercial material used in this study are available from the corresponding author upon reasonable request. Source data are provided with this paper.

## Source data

Source Data
