# Supplementary material for: The IGFBP3/TMEM219 pathway regulates beta cell homeostasis
Source: Nat Commun. 2022 Feb 3;13:684. doi: 10.1038/s41467-022-28360-2 (PMC8813914; doi:10.1038/s41467-022-28360-2)
Supplement: Supplementary file 1 — Supplementary Information [file 41467_2022_28360_MOESM1_ESM.pdf]

SUPPLEMENTARY INFORMATION

The IGFBP3/TMEM219 pathway regulates beta cell homeostasis

Francesca D'Addio[1], Anna Maestroni[1], Emma Assi[1], Moufida Ben Nasr[1,2], Giovanni Amabile[3], Vera Usuelli[1,2], Cristian Loretelli[1], Federico Bertuzzi[4], Barbara Antonioli[4], Francesco Cardarelli[5], Basset El Essawy[6,7], Anna Solini[8], Ivan C. Gerling[9], Cristina Bianchi[10], Gabriella Becchi[11], Serena Mazzucchelli[1], Domenico Corradi[11], Gian Paolo Fadini[12], Diego Foschi[13], James F Markmann[14], Emanuela Orsi[15], Jan Škrha jr[16], Maria Gabriella Camboni[3], Reza Abdi[6], A.M. James Shapiro[17], Franco Folli[18], Johnny Ludvigsson[19], Stefano Del Prato[10], Gian Vincenzo Zuccotti[20] and Paolo Fiorina[1,2,21*]

[1]International Center for T1D, Pediatric Clinical Research Center Romeo ed Enrica Invernizzi, DIBIC, Università di Milano, Milan, Italy; [2]Nephrology Division, Boston Children's Hospital and Transplantation Research Center, Brigham and Women's Hospital, Harvard Medical School, Boston, MA, USA; [3]Enthera S.r.l., Milano, Italy; [4]Diabetology Unit, ASST Grande Ospedale Metropolitano Niguarda, Milan, Italy; [5]NEST-Scuola Normale Superiore, Pisa, Italy; [6]Transplantation Research Center, Nephrology Division, Brigham and Women's Hospital, Boston, MA; [7]Medicine, Al-Azhar University, Cairo, Egypt; [8]Department of Surgical, Medical and Molecular Pathology and Critical Care Medicine, University of Pisa, Pisa, Italy; [9]Department of Medicine, University of Tennessee, Memphis, TN, USA; [10]Section of Diabetes and Metabolic Disease, Department of Clinical and Experimental Medicine, University of Pisa and Azienda Ospedaliero-Universitaria Pisana, Pisa, Italy; [11]Department of Medicine and Surgery, Unit of Pathology, University of Parma, Parma, Italy; [12]Department of Medicine, University of Padua, Padua, Italy; [13]General Surgery, DIBIC, L. Sacco Hospital, Università di Milano, Milan, Italy; [14]Division of Transplantation, Department of Surgery, Massachusetts General Hospital, Harvard Medical School, Boston, MA, USA; [15]Diabetes Service, Endocrinology and Metabolic Diseases Unit, IRCCS Cà Granda - Ospedale Maggiore Policlinico Foundation, Milan, Italy; [16]3rd Department of Internal Medicine, Charles University, First Faculty of Medicine, Prague, Czech Republic; [17]Clinical Islet Transplant Program, Alberta Diabetes Institute, University of Alberta, Edmonton, AB, Canada; [18]Endocrinology and Metabolism, Department of Health Science, Università di Milano, ASST Santi Paolo e Carlo, Milan, Italy; [19]Crown Princess Victoria Children's Hospital and Div of Pediatrics, Dept of Biomedical and Clinical Sciences, Linköping University, Linköping, Sweden; [20]Pediatric Clinical Research Center Romeo ed Enrica Invernizzi, DIBIC, Università di Milano and Department of Pediatrics, Buzzi Children's Hospital, Milan, Italy; [21]Division of Endocrinology, ASST Fatebenefratelli-Sacco, Milan, Italy.

[*]Corresponding author

TABLE OF CONTENTS:

SUPPLEMENTARY METHODS

SUPPLEMENTARY TABLES 1-8

SUPPLEMENTARY FIGURES 1-7

SUPPLEMENTARY REFERENCES

SUPPLEMENTARY METHODS

Human studies

Patients and study design

T1D study

Plasma samples from 30 patients with established type 1 diabetes (T1D) and from 45 patients with new-onset T1D, from 30 patients screened positive for islets autoantibodies ($AutoAb^{+}$) and from 30 healthy volunteers (CTRL) were obtained with informed consent as reported in Supplementary Table 1. T1D were all on intensive insulin treatment at the time of enrollment in the study, while the CTRL and the $AutoAb^{+}$ group were not being administered any medication. All T1D subjects were on the same treatment with regard to antiplatelet therapy (ASA) and anti-hypertension (angiotensin-converting-enzyme inhibitors).

T2D study

235 serum samples from normal glucose tolerant (NGT) patients, 200 serum samples from impaired glucose tolerant (IGT) patients, and 81 serum samples from newly-diagnosed type 2 diabetes (T2D) patients were collected at the University of Pisa (Italy) under the GENFIEV protocol study. NGT, IGT, and T2D classifications were determined based on the results of OGTT tests according to the ADA 2003 criteria. The 844 participants (44% men, 56% women) in the GENFIEV study had an average age of 49.5±11 years (range 22–79) and a BMI of 29±5 kg/m^2 (range 16.5–51). According to the 2003 ADA criteria, 43% of the study population had NGT, 42% had impaired glucose regulation (IGR: IFG and/or IGT), and 15% had newly diagnosed T2D. Analysis of IGFBP3 levels was successfully performed and determined for each group as follows: T2D: n=70/81; IGT: n=149/200;

NGT: n=146/235. Clinical and demographics characteristics are reported in Supplementary Table 2. Concomitant treatment, inclusion and exclusion criteria have been previously described and are reported at the following website https://clinicaltrials.gov/ct2/show/record/NCT00879801?term=GENFIEV [1].

Liver transplantation study

Samples were also obtained from 20 diabetic liver-transplanted patients before transplantation and at 2 years of follow-up [2]. 10 out of 20 patients remained diabetic at the time of 2-year follow-up (non-regressor), while 10 patients near-normalized their glycometabolic control (regressor). Demographic and clinical characteristics are reported in Supplementary Table 3.

Islet transplantation study

Plasma samples were also obtained from 17 islet-transplanted patients with T1D before and after transplantation with a mean ± SEM follow-up of 16.3±1.6 months. Baseline characteristics were as follows (mean ± SEM): HbA1c: 7.3±0.2% (56.5±2.4 mmol/mol); age: 54.5±2.4 years; history of T1D: 41.0±2.7 years. With regard to immunosuppressive treatment, 13 patients were on mycophenolate mofetil plus tacrolimus, 2 patients were on mycophenolic acid delayed release plus tacrolimus, 1 patient was on tacrolimus alone and 1 patient received alemtuzumab with mycophenolate mofetil plus tacrolimus. A measurement of C-peptide < 0.4 ng/ml was considered indicative of a failing islet transplantation.

Obese T2D study

Serum samples were obtained from 18 obese diabetic patients selected for bariatric surgery treatment whose baseline characteristics were as follows (mean ± SEM): HbA1c: 8.0±0.3%

(63.5±3.6 mmol/mol); fasting plasma glucose: 167.5±12.1 mg/dl; age: 49.3±2.6 years; BMI: 40.0±1.5 Kg/m^2. All subjects provided informed consent before study enrollment. Studies not included in the routine clinical follow-up were approved at each Institution (Supplementary Table 4).

Pathology and immunohistochemistry

Samples were fixed in buffered formalin (formaldehyde 4% w/v and acetate buffer 0.05 M) and routinely processed in paraffin wax. 3µm-thick sections of each enrolled case were stained with hematoxylin and eosin (H&E) for morphological evaluation. For immunohistochemistry, 3µm-thick sections were mounted on poly-L-lysine coated slides, deparaffinized and hydrated through graded alcohol to water. After antigen retrieval, performed by dipping sections in 0.01 M citrate buffer, pH 6 for 10 minutes in a microwave oven at 650W as well as endogenous peroxidase activity inhibition, performed by dipping sections in 3% hydrogen peroxide for 10 minutes, incubation with primary antibodies was performed at 4°C for 18–20 h, followed by the avidin-biotin complex procedure. Immunoreactions were developed using 0.03% 3,3'diaminobenzidine tetrahydrochloride, and then sections were counterstained with Harris' hematoxylin.

In vitro studies

Human islets

Electron microscopy

For ultrastructural studies of insulin granules within human pancreatic islets of healthy donors treated with/without IGFBP3 (72 h), samples were fixed for 1 h with 4% paraformaldehyde and 2.5% glutaraldehyde in 125 mM cacodylate buffer, then centrifuged at high speed. The pellet was postfixed (1 h) with 2% OsO4 in 125 mM cacodylate buffer,

was washed, dehydrated and embedded in Epon. Conventional thin sections were collected on uncoated grids, stained with uranyl acetate and lead citrate and examined in a Talos L120C electron microscope (ThermoFisher, Waltham, MA).

Human beta cell lines and Alpha cells

The human beta cell line Blox-5 was a generous gift from Prof. Clayton Mathews, Department of Pathology, Immunology and Laboratory Medicine, University of Florida College of Medicine, Gainesville, FL. Cells were cultured in DMEM with FBS (10%), BSA (0.02%), HEPES (15 mM), Non-essential amino acids (1x), Glucose (1 g/L), penicillin (100 units/mL), and streptomycin (100 μg/mL). Cells were usually seeded into 35 mm wells at a density of 10,000 cells/well. Cells cultured at 37°C in a humidified incubator in 5% CO2 and were passaged at 80% confluency. The human beta cell line EndoC-βH1 (INSERM, Paris) was grown in culture flasks coated with 2μg/ml fibronectin and 1% ECM in DMEM (glucose 1 g/L), BSA fraction V (2% wt/vol), 2-mercaptoethanol (50 μM), nicotinamide (10 mM), transferrin (5.5 μg/mL), sodium selenite (6.7 ng/mL), penicillin (100 units/mL), and streptomycin (100 μg/mL). The cells were cultured at 37°C in a humidified incubator in 5% CO2 and were passaged once every second week.Cells were cultured for 3 days with/without recombinant proteins/Antibodies as described in the *Recombinant proteins and interventional studies* section. Human Alpha cells were purchased from Celprogen (#35002-05) and cultured as per manufacturer's instructions.

Serum IGFBP3 depletion

Endogenous IGFBP3 was depleted from T1D/T2D sera employed in human beta cell line/islets culture using affinity chromatography. In particular the CaptureSelect™ IgG4 (Hu) Affinity Matrix (290010, ThermoFisher Scientific, Waltham, MA) was added to a

chromatographic column (ThermoFisher Scientific, 89868) and coupled with monoclonal anti-human IGFBP3 antibody (Evitria AG, Schlieren, Switzerland) used at a concentration of 10 ug/ml. Serum samples were passed through the column and IGFBP3-depleted sera were collected for *in vitro* experiments.

Caspase 8 activation

A pan-caspase inhibitor (caspase inhibitor Z-VAD-FMK, 20 mM, Promega, Madison, WI), and a Caspase 8-selective inhibitor (Z-IETD-FMK, BD Biosciences, San Jose, CA), were also used *in vitro* in the human beta cell line Blox-5 to confirm the Caspase 8-mediated IGFBP3 effect at concentrations previously described [3].

Secretory granule study

Cell culture

INS-1 E cells [4] were cultured in RPMI 1640 medium containing 11.1 mM D-glucose, 10 mM Hepes, 10 mM L-Glutamine, 100 U/ml penicillin-streptomycin, 10 mM sodium-pyruvate, 50 μM tissue culture grade β-mercaptoethanol (all purchased from ThermoFisher). Cells were maintained in culture in a 37°C humidified incubator (5% CO_2). To generate a stable cell line of INS-1E expressing syncollin-EGFP, cells were transfected with Lipofectamine 2000 reagent as per manufacturer's instructions with syncollin-EGFP plasmid carrying NeoR gene which confers resistance to Geneticin (G418) eukaryotic antibiotics. 48 h after transfection, the medium was replaced with fresh complete medium supplemented with 50 μg/mL G418. After 15 days of selection with antibiotics, a stable polyclonal cell population expressing syncollin-EGFP was obtained. The G418 concentration was then reduced to 10 μg/mL for 1 week. The day prior to microscopy analysis, $5x10^5$ syncollin-EGFP INS-1E cells were seeded on a IbiTreat μ-Dish 35mm,

with high walls and 1.5 polymer coverslip, that was tissue culture-treated, sterilized and fluorescence microscopy suitable (Ibidi, Martinsried, Germany) devoid of G148.

Fluorescence confocal microscopy experiments

Confocal microscopy experiment on syncollin-EGFP-INS-1E cells were performed using a Zeiss LSM 800 laser confocal microscope (Zeiss, Oberkochen, Germany), with a 63x/1.47 N.A. oil objective. To study the live-cell dynamics of syncollin-EGFP-labeled ISGs under different conditions, videos of fluorescent ISGs were acquired by illuminating the sample with a 488-nm laser. Each acquisition consists of a collection of 500 frames (256x256 pixels) at a sampling frequency of 204 ms/frame and with a pixel size of 50 nm. EGFP fluorescence was collected between 500 and 600 nm. Time-lapse acquisitions were analyzed using a custom script working on MATLAB (MathWorks Inc., Natick, Ma) which computes the spatiotemporal correlation function, and extracts an imaging-derived diffusion law of the labelled granules (also named *i*MSD), as described in detail elsewhere [5, 6, 7]. Proper fitting of the iMSD trace provides the diffusion parameters (e.g. the local granule diffusivity). In addition, single-granule trajectory analysis was performed using the TrackMate plugin of ImageJ. In brief, single-granule fluorescence spots were detected by LogDetector while granule trajectories were retrieved by using the Lap Tracker algorithm.

Immunoblotting

Total proteins of purified islets were extracted in Laemmli buffer (Tris–HCl 62.5 mmol/l, pH 6.8, 20% glycerol, 2% SDS, 5% b-mercaptoethanol). 35 ug of total protein was electrophoresed on 7% SDS-PAGE gels and blotted onto nitrocellulose (Schleicher & Schuell, Dassel, Germany). Blots were then stained with Ponceau S. Membranes were blocked for 1 h in TBS (Tris [10 mmol/l], NaCl [150mmol/l]), 0.1% Tween-20, 5% non-

fat dry milk, pH 7.4 at 25° C, incubated for 12 h with a goat polyclonal TMEM219 (Santa Cruz Biotechnology [Dallas,TX], 244404 or 244405) diluted 1:200 or with a monoclonal mouse anti-β-actin antibody (Santa Cruz Biotechnology) diluted 1:500 in TBS–5% milk at 4° C, washed four times with TBS–0.1% Tween-20, then incubated with a rabbit anti-goat (for TMEM219) or rabbit anti-mouse for β-actin, diluted 1:1000 (Santa Cruz Biotechnology) in TBS–5% milk, and finally washed with TBS–0.1% Tween-20. The resulting bands were visualized using enhanced chemiluminescence (SuperSignal; Pierce, Rockford, IL, USA).

Generation of anti-IGFBP3 monoclonal antibody

Monoclonal anti-IGFBP3 antibodies were discovered from naïve human phage-display libraries using a recombinant full length human IGFBP3 (R&D, 8874-B3) as antigen for the screening. Briefly human IGFBP3 (R&D, 8874-B3) was biotinylated and immobilized via streptavidin onto 96-well ELISA plates at 4 °C. After washing and blocking of the wells with BSA, the antibody-phage libraries were added. Tested libraries were first cleared from antibody-phage that bind to Streptavidin (John deKruif, TonLogtenberg Phage Display of Antibodies Encyclopedia of Immunology (Second Edition) 1998, Pages 1931-1934). Those phages that carried an antigen-specific antibody were captured on the plate surface, and antigen-specific phage were amplified by phage infection in E. coli. At the end of the discovery process, each selection output was screened for antigen-specific antibodies. For this purpose, 384 clones were chosen from each selection output and used for production of monoclonal scFv antibodies. These were then tested for specific antigen binding by ELISA. 24 target specific hits were identified which were sequenced. All antibodies showing unique CDRs sequences were cloned into a mammalian scFv-Fc expression

vector. As a result, the scFv was genetically fused to a Fc Fragment of human IgG4. The resulted anti-IGFBP3 scFv-Fc monoclonal antibodies were produced by transient transfection of HEK293 cells, purified by affinity chromatography (Protein A) and re-buffered in PBS. The protein concentration was determined by UV/VIS spectrometry and purity was checked by Coomassie staining. The sequence of the anti-IGFBP3 monoclonal antibody used here, YU139-C01, is reported below.

Antibody (Ab)	VH CDR1	VH CDR2	VH CDR3
YU139-C01	SYGIS (SEQ ID NO:4)	WINTYNGNTNYAQKLQG (SEQ ID NO:11)	DIGYSGSYYSPYYYYGMDV (SEQ ID NO:20)

Ab	VL CDR1	VL CDR2	VL CDR3
YU139-C01	QGDGLRNYFAS (SEQ ID NO:30)	GKNNRPS (SEQ ID NO:37)	NSRDGSGKHLV (SEQ ID NO:46)

Ab	AA of VH
YU139-C01	QVQLVQSGAEVKKPGASVKVSCKASGYTFTSYGISWVRQAPGQGLEWMGWINTYNGNTNYAQKLQGRVTMTTDTSTTTAYMELRSLRSDDTAVYYCARDIGYSGSYYSPYYYYGMDVWGQGTTVTVSS (SEQ ID NO:56)

Ab	AA of VL
YU139-C01	SSELTQDPAVSVALGQTVRITCQGDGLRNYFASWYQQKPGQAPVLVIYGKNNRPSGIPDRFSGSSSGNTASLRIAGAQAEDEADYYCNSRDGSGKHLVFGGGTKLTV (SEQ ID NO:67)

Ab	DNA of VH
YU139-C01	CAGGTCCAGCTGGTGCAATCTGGAGCTGAGGTGAAGAAGCCTGGGGCCTCAGTGAAGGTCTCCTGCAAGGCTTCTGGCTACACCTTTACCAGCTATGGTATCAGCTGGGTGCGACAGGCCCCTGGACAAGGGCTTGAGTGGATGGGATGGATCAACACTTACAATGGTAACACAAACTATGCACAGAAACTCCAGGGCAGAGTCACCATGACCACAGACACATCCACGACCACAGCCTACATGGAGCTGAGG

	AGCCTGAGATCTGACGACACGGCCGTATATTACTGTGCGAGA GATATCGGGTATAGTGGGAGCTACTATTCGCCCTACTACTACT ACGGTATGGACGTCTGGGGCCAAGGGACCACGGTCACCGTCT CCTCA (SEQ ID NO:78)

Ab	DNA of VL
YU139-C01	TCTTCTGAGCTGACTCAGGACCCTGCTGTGTCTGTGGCCTTGG GACAGACAGTCAGGATCACGTGCCAAGGAGACGGCCTCAGA AACTATTTTGCAAGTTGGTACCAGCAGAAGCCAGGACAGGCC CCTGTACTTGTCATCTATGGTAAAAACAACCGGCCCTCAGGG ATCCCAGACCGATTCTCTGGCTCCAGCTCAGGAAACACAGCT TCCTTGAGAATCGCTGGGGCTCAGGCGGAAGATGAGGCTGAC TATTACTGTAATTCCCGGGACGGCAGTGGTAAGCATCTGGTAT TCGGCGGAGGGACCAAGTTGACCGTC (SEQ ID NO:89)

**Constant region**	**AA**
Human IgG4 heavy chain P01861.1	ASTKGPSVFPLAPCSRSTSESTAALGCLVKDYFPEPVTVS WNSGALTSGVHTFPAVLQSSGLYSLSSVVTVPSSSLGTKT YTCNVDHKPSNTKVDKRVESKYGPPCPSCPAPEFLGGPS VFLFPPKPKDTLMISRTPEVTCVVVDVSQEDPEVQFNWY VDGVEVHNAKTKPREEQFNSTYRVVSVLTVLHQDWLNG KEYKCKVSNKGLPSSIEKTISKAKGQPREPQVYTLPPSQE EMTKNQVSLTCLVKGFYPSDIAVEWESNGQPENNYKTTP PVLDSDGSFFLYSRLTVDKSRWQEGNVFSCSVMHEALHN HYTQKSLSLSLGK (SEQ ID NO:97)
Human IgG2 heavy chain P01859	ASTKGPSVFPLAPCSRSTSESTAALGCLVKDYFPEPVTVS WNSGALTSGVHTFPAVLQSSGLYSLSSVVTVPSSNFGTQT YTCNVDHKPSNTKVDKTVERKCCVECPPCPAPPVAGPSV FLFPPKPKDTLMISRTPEVTCVVVDVSHEDPEVQFNWYV DGVEVHNAKTKPREEQFNSTFRVVSVLTVVHQDWLNGK EYKCKVSNKGLPAPIEKTISKTKGQPREPQVYTLPPSREE MTKNQVSLTCLVKGFYPSDISVEWESNGQPENNYKTTPP MLDSDGSFFLYSKLTVDKSRWQQGNVFSCSVMHEALHN HYTQKSLSLSPGK (SEQ ID NO:99)
Human light chain, lambda 1 P0CG04	GQPKANPTVTLFPPSSEELQANKATLVCLISDFYPGAVTV AWKADGSPVKAGVETTKPSKQSNNKYAASSYLSLTPEQ WKSHRSYSCQVTHEGSTVEKTVAPTECS (SEQ ID NO:99)
Human light chain, lambda 2 P0DOY2	GQPKAAPSVTLFPPSSEELQANKATLVCLISDFYPGAVTV AWKADSSPVKAGVETTTPSKQSNNKYAASSYLSLTPEQ WKSHRSYSCQVTHEGSTVEKTVAPTECS (SEQ ID NO:100)
Human light chain, kappa P01834	RTVAAPSVFIFPPSDEQLKSGTASVVCLLNNFYPREAKVQ WKVDNALQSGNSQESVTEQDSKDSTYSLSSTLTLSKADY EKHKVYACEVTHQGLSSPVTKSFNRGEC (SEQ ID NO:101)

Animal studies

Generation of TMEM219$^{flox/flox}$ and Beta-TMEM219$^{-/-}$ mice

The TMEM219$^{flox/flox}$ mouse model was generated using Clustered Regularly Interspaced Short Palindromic Repeats (CRISPR) technology. Two LoxP cassettes were inserted in introns 1-2 and 3-4, flanking exons 2 and 3 of the *mTmem219* gene (Fig. 4p, Supplementary Fig. 6a). When crossed with a mouse expressing Cre, exons 2 to 3 (totaling 420 bp) are deleted upon tamoxifen injection. First, a mixture containing guide RNA molecules (gRNA) and qualified Cas9 mRNA was injected into the cytoplasm of C57BL/6J (B6) embryos. B6 embryos were intracytoplasmically injected with a CRISPR targeting cocktail containing Tmem219.5'g1 and Tmem219.3'g4 for targeting intron 1-2 and intron 3-4. Embryos that developed well *in vitro* were subsequently implanted into CD1 surrogate mice. Newborn mice were screened by PCR and sequencing, and mice were confirmed for successful targeting by assessing the sequence in the modified region following cloning of the PCR product and sequencing of individual clones. Once generated, TMEM219$^{flox/flox}$ mice were mated with homozygous INS^{cre} (B6(Cg)-Ins1tm1.1(cre)Thor/J) purchased from The Jackson Laboratories (Bar Harbor, ME). Experiments were performed using homozygous TMEM219$^{flox/flox}$INS^{cre} male mice (BetaTMEM219$^{-/-}$). In order to demonstrate the effect of TMEM219 genetic ablation on pancreatic beta cells, BetaTMEM219$^{-/-}$ mice were first injected with tamoxifen (Sigma Aldrich T5648, 20 mg/ml, 100 ul at day 0 and day +1 i.p.) to induce Cre-mediated deletion of TMEM219 on insulin-positive cells. BetaTMEM219$^{-/-}$ mice in which tamoxifen was not injected and the Cre/lox system was not activated were used as controls. For intraperitoneal glucose tolerance test (IPGTTs) mice were fasted overnight for approximately 12 hours and then

injected intraperitoneally (i.p.) with glucose 1g/Kg. Blood samples were obtained at the time points 0, 30, 60 and 120 minutes for glucose measurements using a glucometer.

The high-fat diet B6 model

To study the onset and progression of T2D, B6 male mice (7-8 weeks old) were fed with either a high-fat diet (HFD) (DIO diet D12492, 60% of total calories from fat) or a normal-fat diet (NFD; DIO diet D12450B; 10% of total calories from fat), purchased from Research Diets (Mucedola, Settimo Milanese, Italy) for 12 weeks. Blood glucose was monitored twice per week for up to 12 weeks in order to confirm diabetes onset.

STZ-induced diabetes studies

In order to demonstrate the effect of TMEM219 genetic ablation on pancreatic beta cells, BetaTMEM219$^{-/-}$ mice were first injected with tamoxifen (Sigma Aldrich T5648, 20 mg/ml, 100 ul at day 0 and day +1 i.p.) to induce Cre-mediated deletion of TMEM219 on insulin-positive cells. Next, diabetes was chemically induced by injecting low dose streptozotocin (40 mg/Kg, administered i.p.; Sigma Aldrich S0130) for 5 consecutive days, and glycemia was monitored for the next 20 days. A control group consisting of wild-type B6 mice was injected with low dose streptozotocin as well and monitored accordingly. Low dose streptozotocin injection was also employed in wild type B6 mice (40 mg/Kg for 5 consecutive days), in which ecto-TMEM219 was also administered i.p. from day 0 to day 10 and glycemia was monitored for the following 10 days.

Prevention and reversal studies in NOD mice

Female NOD mice were injected at 10 weeks of age with ecto-TMEM219 at a dose of 0.1 mg/mouse/day for 10 days (short-term) and 0.1 mg/mouse/day for 10 days and twice per week for 10 weeks (long-term) and monitored until week 24. Female NOD mice were

monitored beginning at 10 weeks of age, and after 24 hours of hyperglycemia (>250 mg/dl) were injected with ecto-TMEM219 at a dose of 0.1 mg/day for 10 days or left untreated. Mice were monitored daily by measuring blood glucose until they became terminally hyperglycemic and then were sacrificed.

Histology and immunohistochemistry

Immunohistochemistry was performed with 5-μm-thick formalin-fixed, paraffin-embedded tissue section. Photomicrographs (40X) were taken using an Olympus BX41 microscope (Olympus, Center Valley, PA). Pancreas sections were stained with H&E and polyclonal guinea-pig anti-insulin (1:500; A0564 DAKO/Agilent). A score of 0 to 4 was assigned based on islet area and infiltrating cells detected by an experienced pathologist, as previously described[8]. At least 25 islets per group were analyzed and pooled from sections obtained from different mice.

Immunological studies

Isolation of splenocytes and flow cytometric staining

Lymphocytes were isolated from spleens of ecto-TMEM219-treated, anti-TMEM219-treated and untreated NOD mice as previously described [9]. Briefly, each spleen was mashed, and pooled single-cell leukocyte suspensions were prepared for flow cytometric staining. Anti-mouse CD4 (clone H129.19), CD8 (clone 53-6.7), CD25 (clone: PC61), CD44 (clone: IM7), CD62L (clone: MEL-14), and FoxP3 (MF23) were purchased from BD Biosciences (553651, 563786, 564022, 553134, 740660 and 560408), (1:100 diluition). Flow cytometric analysis was performed using a BD FACS Celesta flow cytometry system (BD Biosciences) and analyzed using FlowJo software (version 6 or version 10, Tree Star, Ashland, OR).

Enzyme-linked immunospot assay (ELISpot)

An ELISpot assay was used to measure the number of IFN-γ-producing cells according to the manufacturer's instructions (552569, BD Biosciences). $1x10^6$ splenocytes obtained from ecto-TMEM219-treated, anti-TMEM219-treated and untreated NOD mice were cultured for 24 h in the presence of the BDC2.5 or in the presence of the IGRP murine islet peptide (150µg/ml, AS-63774, AnaSpec, Fremont, CA). Spots were counted using an ImmunoSpot® ELISPOT Reader (CTL Europe GmbH, Bonn, Germany).

SUPPLEMENTARY TABLES

Supplementary Table 1. Baseline demographic characteristics of human subjects enrolled for the study of IGFBP3 levels as shown in Figure 2 with regard to type 1 diabetes (T1D).

	**CTRL (n=30)**	**T1D (n=30)**	**AutoAb$^+$ (n=30)**	**New-onset T1D (n=45)**	***p value***
Age (years)	30.8 ± 1.6	34.7 ± 2.1#	19.5 ± 2.4§	10.4 ± 0.4°	#ns<br>°<0.0001<br>§<0.0001
History of T1D	-	22.3 ± 3.8 years	-	10 days	-
HbA1c % (mmol/mol)	5.0 ± 0.05 (31.0 ± 0.5)	9.2 ± 0.4 (78.0 ± 4.0)	-	N/A	#<0.0001
Fasting C-peptide (nmol/l)	-	< 0.03	-	0.2 ± 0.03	-
EIR (UI)	-	42.0 ± 4.1	-	-	-

#p value CTRL vs. T1D; °p value new-onset T1D vs. CTRL; §p value CTRL vs. AutoAb$^+$ by One-way ANOVA followed by Bonferroni's post-hoc test.

Abbreviations. CTRL, healthy volunteers; T1D, type 1 diabetic patients; AutoAb$^+$, subjects positive for autoantibodies; New-onset T1D, subjects with onset of type 1 diabetes within 10 days; HbA1c %, glycated hemoglobin A1c %; EIR, exogenous insulin requirement; N/A, not applicable; ns, not significant. Data are expressed as mean ± standard error of mean (SEM).

Supplementary Table 2. Baseline demographic characteristics of human subjects enrolled for the study of IGFBP3 levels as shown in Figure 2 with regard to type 2 diabetes (T2D).

	**NGT (n=146)**	**IGT (n=149)**	**T2D (n=70)**	***p value***
Age (years ± SEM)	43.8 ± 12.5	52.4 ± 9.4	55.1 ± 8.4	*<0.0001; °<0.0001; ^ns
HbA1c (%)	5.4 ± 0.03	5.7 ± 0.03	6.2 ± 0.08	*°^<0.0001
OGTT baseline (mg/dl ± SEM)	87.2 ± 0.6	102.3 ± 0.8	120.4 ± 2.1	*°^<0.0001
BMI (Kg/m^2)	27.9 ± 5.0	29.1 ± 4.5	29.5 ± 5.9	*0.03; °0.02; ^ns
SBP systolic pressure (mmHg ± SEM)	122.1 ± 1.1	133.3 ± 1.3	136.1 ± 2.1	*<0.0001; °<0.0001; ^ns
DBP diastolic pressure (mmHg ± SEM)	76.2 ± 1.0	83.3 ± 0.9	84.5 ± 1.4	*<0.0001; °<0.0001; ^ns
Triglycerides (mg/dl ± SEM)	115.1 ± 7.9	149.4 ± 8.4	184.2 ± 16.9	*0.009; °<0.0001; ^0.02

Abbreviations. NGT, patients with normal glucose tolerance; IGT, patients with impaired glucose tolerance; T2D, type 2 diabetic patients; HbA1c %, glycated hemoglobin A1c %; OGTT, oral glucose tolerance test; BMI, body mass index; SBP, systolic blood pressure; DBP, diastolic blood pressure; N/A, not applicable; ns, not significant. Data are expressed as mean ± standard error of mean (SEM); *p value NGT vs. IGT; °p value NGT vs. T2D; ^p value IGT vs. T2D by One-way ANOVA followed by Bonferroni's post-hoc test.

Supplementary Table 3. Baseline demographic characteristics of diabetic liver-transplanted patients who reverted to diabetes (regressor) or were free from disease (non regressor) after transplantation at 24 months of follow-up (Figure 2).

	**Liver-Tx recipients regressor (n=10)**	**Liver-Tx recipients non regressor (n=10)**	***p value***
Age (years ± SEM)	59.2 ± 1.7	56.2 ± 2.0	Ns
Hepatic disease	Infection (n=7)<br>Alcoholic (n=1)<br>Metabolic (n=1)<br>Autoimmune (n=1)	Infection (n=8)<br>Alcoholic (n=1)<br>Metabolic (n=1)	-
OGTT baseline (mg/dl ± SEM)	90.1 ± 2.4	113.8 ± 3.5	<0.0001
OGTT 120 min (mg/dl ± SEM)	130.1 ± 6.8	253.6 ± 11.8	<0.0001
Immunosuppressive treatment	FK + MMF (n=4)<br>CsA + MMF (n=5)<br>MMF (n=1)	FK + MMF (n=4)<br>CsA + MMF (n=3)<br>MMF (n=1)<br>CsA (n=1)<br>FK (n=1)	-
Anti-diabetic therapy	None	Metformin + insulin (n=4)<br>Insulin (n=5)<br>Diet (n=1)	-

Abbreviations. Tx, patients who received liver transplantation; OGTT, oral glucose tolerance test; min, minutes; ns, not significant; FK, tacrolimus; CsA, cyclosporine; MMF, mycophenolate mofetil. Data are expressed as mean ± standard error of mean (SEM); *p value Regressor vs. Non regressor by two-sided t-test followed by Welch correction.

Supplementary Table 4. Description of the human cohorts' clinical studies.

Clinical study	Inclusion criteria	Exclusion criteria	Type of study	Informed consent
T1D Study ImmunoT1D	- type 1 diabetes<br>- risk of developing diabetes (positive screening for autoantibodies)<br>- no diabetes.	major surgery<br>active infections,<br>history of malignancy<br>pregnancy<br>breastfeeding<br>type 2 diabetes	Observational<br>Cohort<br>Cross-sectional	Yes
Role of TMEM219 Marker in Type 1 Diabetes (NPOD) (NCT03794739)	- diabetes (type 1, type 2, MODY)<br>- at risk of developing diabetes (positive screening for autoantibodies)<br>- no diabetes	not willing to give consent	Repository	Yes
T2D Study Genetic Physiopathology and Evolution of Type 2 Diabetes (GENFIEV) (NCT00879801)	- IGT (FPG < 7.0 mmol/l, and 2-h PG > 7.8 and < 11.1 mmol/l)<br>- IFG (FPG > 6.1 and < 7.0 mmol/l, and 2-h PG < 11.1 mmol/l)<br>- IFG and IGT<br>- normal glucose tolerance	pregnancy,<br>breastfeeding<br>active arterial disease<br>history of malignancy<br>uncontrolled hypertension<br>hypothyroidism<br>alcohol/drug abuse<br>active liver disease	Observational<br>Cohort<br>Prospective	Yes
Liver Tx Study: Cirrhosis, Effects of Transplantation and Diabetes (CETRA) (NCT02038517)	- end-stage liver cirrhosis in waiting list for orthotopic liver transplantation	known diabetes<br>cystic fibrosis<br>pregnancy	Observational<br>Cohort<br>Prospective	Yes
Islet Transplantation Study	- type 1 diabetes who received islet transplantation	active infections,<br>history of malignancy<br>pregnancy<br>breastfeeding<br>type 2 diabetes	Observational<br>Cohort<br>Prospective	Yes
Obese type 2 diabetes Study	-type 2 diabetes and BMI > 29 kg/m^2 undergoing bariatric surgery	major surgery<br>active infections,<br>history of malignancy<br>pregnancy	Observational<br>Cohort<br>Prospective	Yes
Cadaveric purified human islets Study	-healthy donors whose pancreata was not suitable for organ donation	active infections,<br>history of malignancy<br>diabetes	Repository	Yes

Abbreviations: FPG, fasting plasma glucose; IGT, impaired glucose tolerance; IFT, impaired fasting glucose tolerance

Supplementary Table 5. List of differentially regulated apoptosis-related genes identified by transcriptome profiling in human islets cultured with IGFBP3, with IGFBP3 in the presence of ecto-TMEM219 or left untreated (Figure 3).

	**Downregulated genes**	**Upregulated genes**
**Human islets untreated vs. IGFBP3-treated**		*CASP8, TP53BP2, BCL2L2 TNFRSF10B , CASP9 , TP73, BRAF, BNIP3L, BAG3, TNFSF10, XIAP, BIRC2, CRADD , AIFM1 , BIRC3, FAS, BNIP3, TRAF3, BCL2L10*
**Human islets IGFBP3 treated vs. IGFBP3+Ecto-TMEM219-treated**	*CASP8, TNFSF8, BCL2A1, RIPK2, BNIP3L, FAS, MCL1*	*TNFRSF25, TNFSF8, CASP14*

Abbreviations: IGFBP3, insulin-like growth factor binding protein 3.

Supplementary Table 6. List of differentially regulated beta cell-related genes identified by transcriptome profiling in human islets cultured with IGFBP3, with IGFBP3 in the presence of ecto-TMEM219 or left untreated (Figure 3).

	**Downregulated genes**	**Upregulated genes**
**Human islets untreated vs. IGFBP3-treated**	*INS, PTPN1*	*IRS1, MAPK1, FBP1, PIK3R2, IGF2*
**Human islets IGFBP3 treated vs. IGFBP3+Ecto-TMEM219-treated**	*MAP2K1, SLC2A1, MAPK1, JUN*	*AEBP1*

Abbreviations: IGFBP3, insulin-like growth factor binding protein 3.

Supplementary Table 7. Overview of the available human studies on the role of IGF-I/IGFBP3 axis in type 1 and type 2 diabetes.

Publication (year)	Study population	IGF-I	IGFBP3	Method	Notes
Rajpathak SN et al. [10] (2012) IF: 8	742 T2D<br>742 CTRL<br>women<br>prospective<br>Age: 56 y	→	↑↑	ELISAs (Diagnostic Systems Laboratories)	↑ IGFBP3 is associated with risk of T2D; IGF-I varies with insulin
Bereket A et al. [11] (1995) IF: 6.3	T1D children	N/A	N/A	Immuno-radiometric assay	IGFBP3 levels compared in T1D +/-insulin
Drogan D et al. [12] (2016) IF: 5	776 T2D<br>2269 enrolled for follow up<br>prospective<br>Age: 40-60 y	→	↑	enzyme-linked immunosorbent assay (BioVendor Laboratorní medicína CR)	↑IGFBP3 is associated with risk of T2D also adjusting for IGF-I; no IGF-I correlation.
Aneke-Nash CS et al. [13] (2015) IF: 4.2	166 T2D<br>399 CTRL<br>332 IGT<br>Age: 76 y	↓	↓	ELISA (DSL, Webster, TX)	↓ IGFBP3 and IGF-I in T2D at baseline
Peet A et al. [14] (2015) IF: 3.6	40 T1D with AutoAb<br>80 CTRL<br>Age: 0-3 y	→	↑	Enzyme-labeled chemiluminescent immunometric assay IMMULITE	↑ IGFBP3 in high-risk T1D before seroconversion
Frystyk J et al. [15] (1999) IF: 3.5	26 lean CTRL<br>24 obese CTRL<br>29 obese+T2D<br>7 T1D				
Gutefeldt K et al. [16] (2018) IF: 3.4	605 T1D<br>533 CTRL	↓	↓	Automated chemiluminescent immunoassay (IDS, England) IMMULITE	↑ IGFBP1<br>↓ IGFBP3<br>↓IGF-I in T1D (insulin therapy)
Munoz MT et al. [17] (1996) IF: 2.8	92 T1D<br>600 CTRL<br>Age: 1-18 y	↓	→	RIA (Mediagnost GmbH, Germany)	↓ IGF-I in T1D with puberal stage; IGFBP3 no correlation
Rajpathak SN et al. [18]	158 T2D<br>294 IGT	→	→	enzyme-linked immunosorbent	↓ IGFBP1 in IGT; no clear

(2008) IF: 1.3	470 NGT<br>Age: >65 y			assay (Diagnostics Systems Lab, TX)	association for IGF-I or IGFBP3
Cinaz P et al. [19] (1996) IF: 0.7	24 T1D<br>26 CTRL<br>Age: children prebuberal stage	↓	↓	Immuno-radiometric assay and RIA	↓ IGFBP3 due to proteolytic activity
Kim MS et al. [20] (2014) IF: 0.7	78 T1D<br>47 CTRL<br>Age: children	↓	↑→	N/A	IGFBP-3 positive correlation with HbA1c
Wedrychowicz A et al. [21] (2005) IF: N/A	24 T1D<br>24 T1D + MA<br>17 CTRL<br>Age: 17-19 y	→	↑<br>$p>0.05$	IGFBP3: immuno-radiometric assay (DSL, USA); IGF-I: RIA (Bio-Source)	↑IGFBP3 in T1D without MA (p=ns); no alteration in IGF-I
Dunger DB et al. (2005) IF: N/A	T1D children and adolescents	↓	↓	N/A	Review
Kim MS et al. [22] (2015) IF: N/A	T2D, T1D	N/A	N/A	N/A	Review

IGFBP3, insulin-like growth factor binding protein 3; IGF-I, insulin-like growth factor 1; T1D, type 1 diabetes; T2D, type 2 diabetes; RIA, radioimmunolabelling assay; CTRL, healthy subjects; MA, microalbuminuria; y, years; IGT, impaired glucose tolerance fasting and non-fasting; AutoAb, autoantibodies; IGT, impaired glucose tolerant; NGT, normal glucose tolerant; IF, journal impact factor.

Supplementary Table 8. Main characteristics of primers used in qRT-PCR analysis.

Gene Symbol	UniGene #	Refseq Accession #	Band Size (bp)	Reference Position
Human				
INS	Hs.272259	NM 000207.2	126	252
IGF-IR	Hs.643120	NM_000875.3	64	2248
TMEM219	Hs.460574	NM 001083613.1	60	726
INS1R	Hs.465744	NM_000208.3	91	2270
GLP1-R	Hs.351883	NM 002062.4	78	346
IL1-R	Hs.701982	NM 000877.3	97	1168
SLC2A2	Hs.167584	NM_000340.1	65	1377
GLT-1	Hs.502338	NM 001195728.2	58	1518
EGFR	Hs.488293	NM_005228.3	57	875
BACE2	Hs.43047	NM 012105.4	69	1083
TMEM27	Hs.129614	NM_020665.5	67	464
EPHA	Hs.89839	NM 005232.4	66	238
SLC2A1	Hs.473721	NM 006516.2	76	1224
LRP1	Hs.162757	NM_002332.2	64	656
TGFbR1	Hs.494622	NM 001130916.1	73	646
TGFbR2	Hs.604277	NM_001024847.2	70	1981
CASP8	Hs.599762	NM 001080124.1	124	648
ACTB	Hs.520640	NM_001101	174	730
Murine				
Ins	Mm.4626	NM_008386.3	80	533
Tmem219	Mm.248646	NM_026827,1	78	677
Lrp1	Mm.271854	NM_032538.2	104	2995
TgfbR1	Mm.197552	NM_009370.2	85	90
TgfbR2	Mm.172346	NM_033397.3	132	1656
Casp8	Mm.336851	NM_001080126.1	96	1525
Gapdh	Mm. 304088	NM_008084.2	107	75

Abbreviations: Refseq, reference sequence.

SUPPLEMENTARY FIGURES

Supplementary Figure 1. The death receptor TMEM219 is expressed in beta cells.

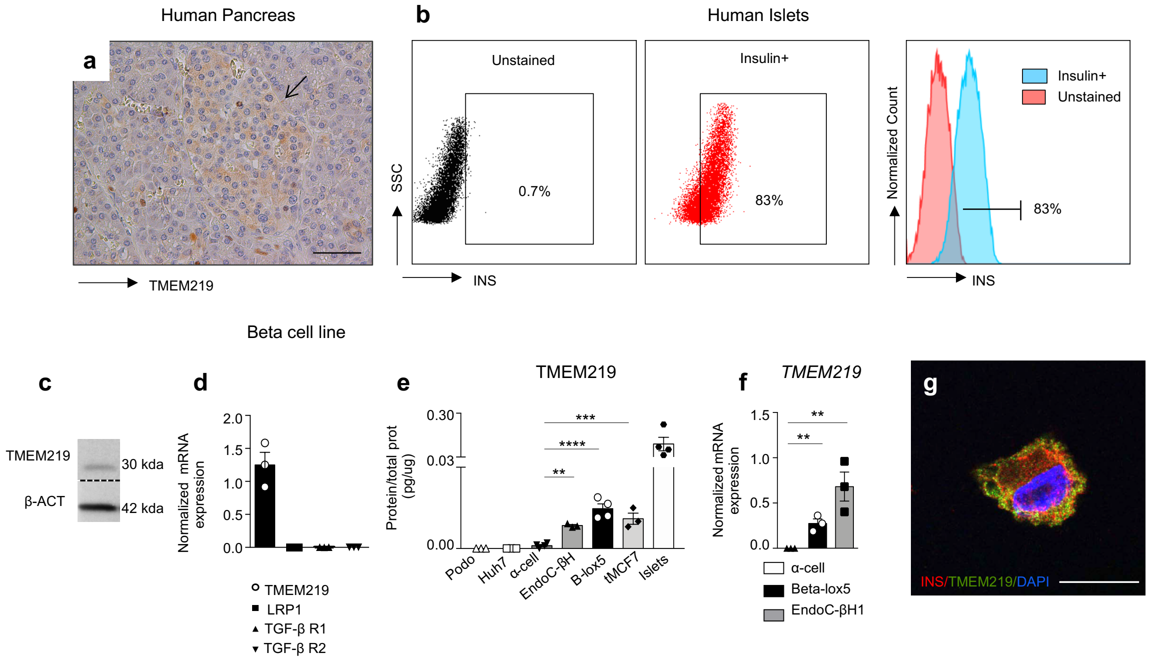

(a). Representative picture of TMEM219 detection by immunohistochemistry in human pancreas (n=3 samples were analyzed). 40X, scale bar 25 μm. **(b)**. Gating strategy showing insulin flow cytometric staining on dissociated human islets. Left plot, unstained; central plot, stained with insulin; histogram, overlayed. **(c)**. Representative cropped immunoblots of TMEM219 protein expression in the human beta cell line. The data shown are the result of three independent experiments. **(d)**. Bar graph depicting gene expression of *TMEM219* and other IGFBP3 receptors (*LRP1*, *TGF-β R1* and *R2*) quantified by qRT-PCR in human beta cell line. The experiment was performed in triplicate. **(e)**. Quantification of TMEM219 protein expression detected by ELISA in cultures of human islets, human beta cell lines and alpha cells. Immortalized podocytes (Podo), Hepatocytes derived cell line (Huh7) were used as negative controls and TMEM219-transfected breast cancer cell line (tMCF7) was

used as positive control. At least n=3 biologically independent experiments were performed. **(f)**. Quantification of TMEM219 mRNA expression by qRT-PCR in human beta and alpha cell lines. At least n=3 biologically independent experiments were performed. **(g)**. Confocal microscopy imaging (scale bar 5 µm, 63X original magnification) depicting baseline co-localization of TMEM219 (green) and insulin (red) in a human beta cell line. Cells were stained with DAPI (blue) and immunolabeled with anti-TMEM219 (green) and anti-insulin Abs (red). The data shown are the result of three independent experiments. Data are expressed as mean ± standard error of the mean (SEM) unless otherwise reported. mRNA expression was normalized to β-actin (*ACTB*). $^{**}p<0.01$, $^{***}p<0.001$, $^{****}p<0.0001$ by One-way ANOVA followed by Bonferroni´s post hoc test. In c-f EndoC-βH1 were used, Beta-lox5 were used in f.

Abbreviations: qRT-PCR, quantitative real-time polymerase chain reaction; SSC, side scatter; SEM, standard error of mean.

Supplementary Figure 2. The IGFBP3/TMEM219 axis is dysregulated in diabetes.

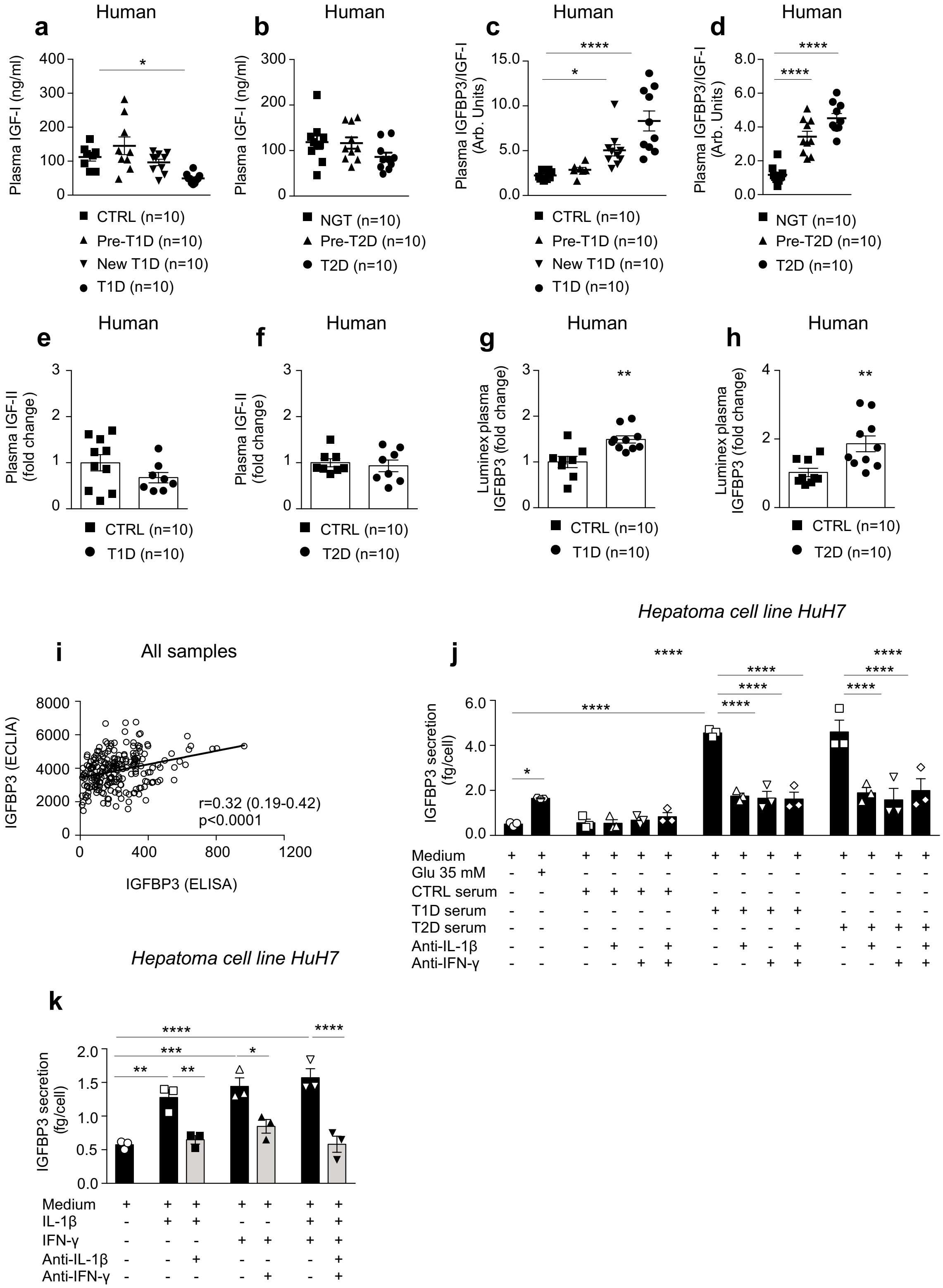

(a). Scatter plots representing peripheral IGF-I levels measured in plasma obtained from patients (n=10/group) with established and new-onset T1D, at risk for developing T1D (with at least one detected autoantibody, pre-T1D) and in healthy controls (CTRL). Demographic characteristics are reported in Supplementary Table 1. **(b)**. Scatter plots depicting peripheral IG-I levels measured in plasma obtained from patients (n=10/group) with T2D, at risk for developing T2D (with altered glucose tolerance, pre-T2D) and in healthy controls (with normal glucose tolerance, NGT). Demographic characteristics are reported in Supplementary Table 2. **(c**, **d)**. Scatter plots showing the IGFBP3/IGF-I plasma ratio in T1D (c) and T2D (d) groups. **(e**, **f)**. Scatter plots representing peripheral IGF-II level (fold change) measured in plasma obtained from patients (n=10/group) with established T1D as compared to healthy controls (CTRL, e), and in patients with T2D as compared to subjects with normal glucose tolerance (NGT, f). Demographic characteristics are reported in Supplementary Table 1 and 2. **(g**, **h)**. Scatter plots showing the increase in IGFBP3 plasma levels (fold change) in established T1D patients as compared to healthy controls (g) and in patients with T2D as compared to subjects with NGT (h) assessed by Luminex. Demographic characteristics are reported in Supplementary Table 1 and 2. **(i)**. Line graph depicting the positive correlation established between IGFBP3 levels measured by ELISA and IGFBP3 levels measured by a clinically grade electrochemiluminescence assay (ECLIA) in the samples obtained within the study groups. Pearson correlation analysis is shown. **(j)**. Bar graphs depicting secreted IGFBP3 measured in hepatocytes derived cell line (Huh7) cultured with regular medium containing 10% FBS and 5 mM glucose, or regular medium with high glucose (35 mM), or medium containing CTRL or T1D/T2D serum in place of FBS 10% with/without neutralizing IFN-γ and/or IL-1β

antibodies. (n=3 independent experiments were performed in duplicate). IGFBP3 levels were obtained by subtracting IGFBP3 level detected in culturing medium added with 10% CTRL/T1D/T2D serum from the total levels of IGFBP3 measured in each condition. **(k)**. Bar graphs representing secreted IGFBP3 measured in hepatocytes derived cell line cultured with regular medium containing 10% FBS and 5 mM glucose, or exposed to a diabetogenic pro-inflammatory environment (IL-1β, IFN-γ or IL-1β+IFN-γ) in the presence/absence of neutralizing IFN- γ and/or IL-1β antibodies (anti-IL-1β, anti-IFN-γ). (n=3 independent experiments were performed in duplicate). Data are expressed as mean ± standard error of the mean (SEM) unless otherwise reported. mRNA expression was normalized to β-actin (*ACTB*). All parameters examined were significantly statistically different when comparing different groups as follows: *$p<0.05$, **$p<0.01$, ***$p<0.001$, ****$p<0.0001$ by One-way ANOVA followed by Bonferroni´s post hoc test, Kruskal-Wallis test adjusted for multiple comparison, two-sided Mann–Whitney U-test. Pearson’s correlation is presented in panel i.

Abbreviations: T1D, type 1 diabetes; pre-T1D, patients positive to at least one autoantibody; AutoAb, autoantibodies; new T1D, new-onset T1D; T2D, type 2 diabetes; NGT, normal glucose tolerance; IGT, impaired glucose tolerance; Glu, glucose; IL-1β, interleukin beta; IFN-γ, interferon gamma; Arb. units, arbitrary units; SEM, standard error of mean.

Supplementary Figure 3. **The IGFBP3/TMEM219 signaling alters beta cell function**.

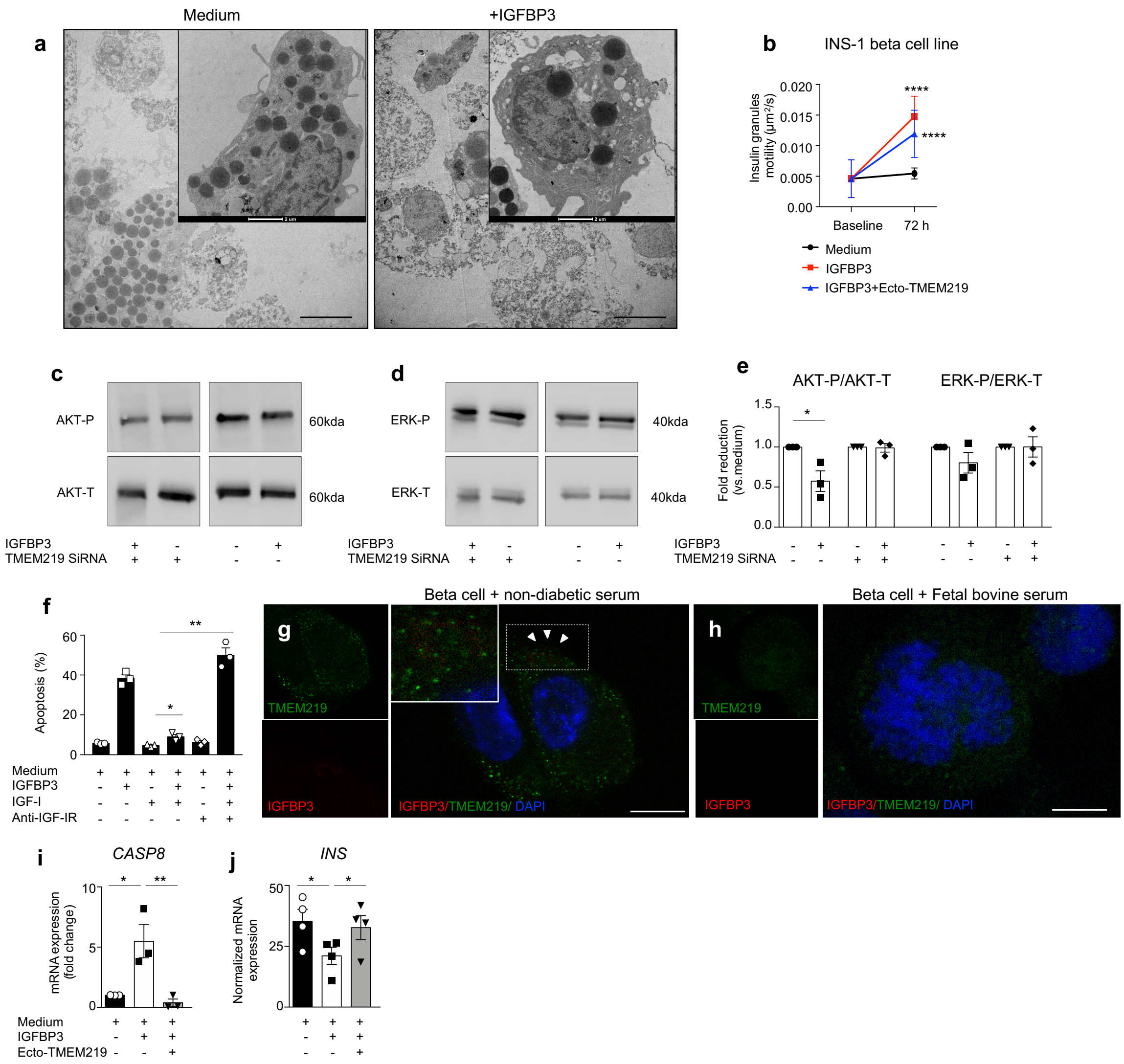

(a). Representative images showing number and size of insulin granules detected in human purified islets of healthy donors cultured with/without IGFBP3, using electron microscopy (n=3). 2000X and 5300X original magnification, respectively; scale bar: 5 and 2 μm, respectively. **(b)**. Line graph depicting the local diffusivity/motility of insulin granules in a beta cell line (INS-1E) stably transfected with an EGFP-tagged variant of proinsulin and analyzed using spatiotemporal fluorescence fluctuation spectroscopy on live-cell time-lapse acquisitions at baseline and after culture with or without IGFBP3 and in the presence

or absence of ecto-TMEM219 (72 h). Experiments were performed in triplicate. **(c**, **d)**. Representative cropped immunoblots showing expression of total AKT and phosphorylated AKT (c) and total ERK1/2 and phosphorylated ERK1/2 (d) analyzed in human islets cultured with/without IGFBP3 or in which TMEM219 signaling was blocked with siRNA in the presence/absence of IGFBP3 (n=3 samples/group). Analysis is reported in Figure 3r. **(e)**. Quantification of activated AKT and ERK1/2 analyzed in human islets cultured with/without IGFBP3 or in which TMEM219 signaling was blocked with siRNA in the presence/absence of IGFBP3 (n=3 samples/group). **(f)**. Bar graph showing apoptosis (% of $Apoptag^{+}INS^{+}$ staining) in human purified islets cultured with/without IGFBP3 and in the presence/absence of IGF-I and/or anti-IGF-IR antibody (n=3). **(g**, **h)**. Representative picture of confocal microscopy analysis (scale bar 10 μm, 63X original magnification) depicting colocalization of TMEM219 (green) and serum IGFBP3 (red) in a human beta cell line cultured with non-diabetic serum (g) and FBS (h). Cells were stained with DAPI (blue) and immunolabeled with anti-TMEM219 (green) and anti-IGFP3 Abs (red). (**i**, **j**). Bar graph depicting normalized mRNA expression of *CASP8* and *INS* quantified by qRT-PCR in human purified islets cultured with/without IGFBP3 and in the presence/absence of ecto-TMEM219 (n=4). Data are expressed as mean ± standard error of the mean (SEM) unless otherwise reported. *p<0.05; **p<0.01; ***p<0.001; ****p<0.0001 by Two-way ANOVA, Kruskal-Wallis adjusted for multiple comparison and by One-way ANOVA with Bonferroni's post-hoc test. mRNA expression was normalized to *ACTB*. The data presented in a, c, d, g and h are the results of three independent experiments.

Abbreviations: INS, insulin; CASP8, Caspase 8; ecto-TMEM219, newly generated recombinant protein based on TMEM219 extracellular portion.

Supplementary Figure 4. The IGFBP3/TMEM219 axis promotes beta cell damage via Caspase 8-mediated apoptosis.

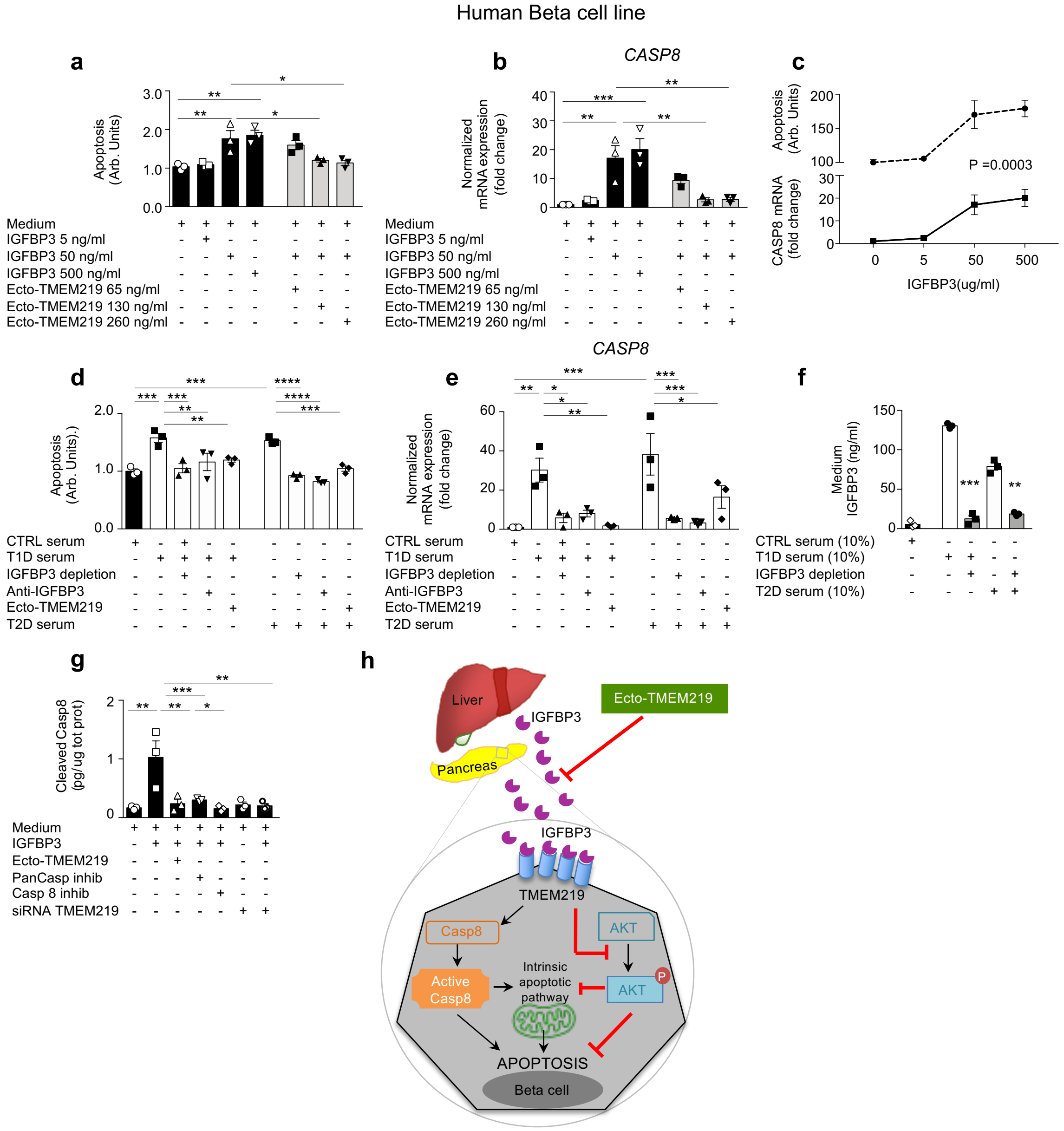

(a). Quantitative bar graphs showing apoptosis analyzed in a human beta cell line (B-lox5) cultured with IGFBP3 at the following dilutions 5, 50 and 500 ng/ml and in the presence or absence of the IGFBP3 inhibitor ecto-TMEM219 at the following molar ratio vs. IGFBP3, 1:2 (65 ng/ml), 1:1 (130 ng/ml), 2:1 (260 ng/ml), (n=3 separate experiments,

performed in duplicate). **(b)**. Bar graphs representing *CASP8* normalized expression (fold change) quantified by qRT-PCR in a human beta cell line cultured with IGFBP3 at the following dilutions 5, 50 and 500 ng/ml and in the presence or absence of the IGFBP3 inhibitor ecto-TMEM219 at the following molar ratio vs. IGFBP3, 1:2 (65 ng/ml), 1:1 (130 ng/ml), 2:1 (260 ng/ml), (n=3 separate experiments, performed in duplicate). **(c)**. Line graph depicting apoptosis and CASP8 mRNA relative expression measured in B-lox5 exposed to increasing IGFBP3 concentrations. The graph shows a significant correlation between increase in apoptosis and in *CASP8* expression (n=3 separate experiments, performed in duplicate). **(d)**. Quantitative bar graphs showing apoptosis analyzed in a human beta cell line cultured with T1D/T2D serum or with CTRL serum pooled from n=5 subjects in place of regular FBS 10%, exposed to IGFBP3 depletion, or supplemented with anti-IGFBP3 monoclonal antibody, or in the presence or absence of the IGFBP3 inhibitor ecto-TMEM219, (n=3 separate experiments, performed in duplicate). **(e)**. Bar graphs representing *CASP8* normalized expression (fold change) quantified by qRT-PCR in a human beta cell line cultured with T1D/T2D serum or with CTRL serum in place of regular FBS 10%, exposed to IGFBP3 depletion, or supplemented with anti-IGFBP3 monoclonal antibody, or in the presence or absence of the IGFBP3 inhibitor ecto-TMEM219, (n=3 separate experiments, performed in duplicate). **(f)**. Levels of IGFBP3 measured in culturing medium in which 10% of T1D/T2D serum pooled from n=5 patients, was added in place of regular FBS and IGFBP3 has been removed or not through immune depletion. Experiment was performed in triplicates. **(g)**. Bar graphs quantifying Cleaved Caspase 8 by ELISA in a human beta cell line (B-lox5) cultured with/without IGFBP3 and in the presence/absence of ecto-TMEM219, and/or in the presence/absence of Caspase

8/Pancaspase inhibitors, or in which TMEM219 was targeted through siRNA, compared to islets in which TMEM219 targeting was not performed (n=3 separate experiments). **(h)**. Schematic representation of the proposed mechanisms whereby circulating IGFBP3 impairs TMEM219-expressing beta cells, which can be counteracted by pharmacological blockade of the IGFBP3/TMEM219 pathway. Data are expressed as mean ± standard error of the mean (SEM) unless otherwise reported. *$p<0.05$; **$p<0.01$; ***$p<0.001$, ****$p<0.0001$ by One-way ANOVA with Bonferroni's post-hoc test, Pearson correlation analysis (c). mRNA expression was normalized to β-actin (ACTB). Apoptosis was normalized to medium in a and to CTRL serum in d.

Abbreviations: CTRL, healthy volunteers' serum; Arb. units, arbitrary units; CASP8, Caspase 8; ecto-TMEM219, newly generated recombinant protein based on TMEM219 extracellular portion; anti-IGFBP3, anti-IGFBP3 depleting monoclonal antibody. T1D, type 1 diabetes; T2D, type 2 diabetes; INS, insulin; Casp 8, Caspase 8; inh, inhibitor; siRNA, small interfering RNA.

Supplementary Figure 5. **Pharmacological targeting of the IGFBP3/TMEM219 axis *in vivo* in preclinical models of diabetes preserves beta cell mass**.

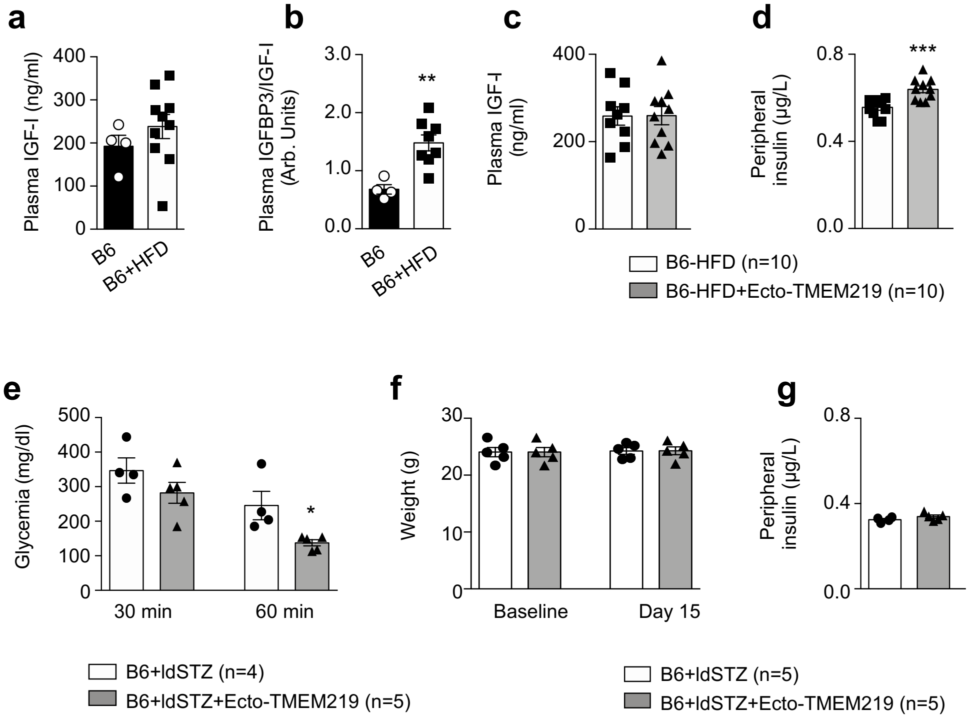

(a). Bar graph showing peripheral IGF-I levels measured in plasma obtained from 8-week-old B6 mice fed a high-fat diet (B6-HFD, n=10) or left untreated (B6, n=5). **(b)**. Bar graphs representing IGFBP3/IGF-I plasma ratio in B6-HFD, (n=10) as compared to B6 fed a regular diet (n=5). **(c**, **d)**. Bar graph showing peripheral IGF-I (c) and insulin (d) levels measured in plasma of B6 mice fed a high-fat diet and treated with ecto-TMEM219 (B6-HFD-ecto-TMEM219, n=10) or left untreated (B6-HFD, n=10). **(e)**. Bar graph showing IPGTT blood glucose levels measured at 30 and 60 minutes in ldSTZ-B6 mice treated with ecto-TMEM219 or left untreated (n=4-5). **(f)**. Bar graph showing body weight measured at baseline and at the end of the study (day 15) in ldSTZ-B6 mice treated with ecto-TMEM219 or left untreated (n=4-5). **(g)**. Bar graph showing plasma insulin levels measured at the end of the study (day 15) in ldSTZ-B6 treated with ecto-TMEM219 or left

untreated (n=5). Data are expressed as mean ± standard error of the mean (SEM) unless otherwise reported. *p<0.05; **p<0.01; ***p<0.001; ****p<0.0001 by two-sided Mann–Whitney U-test, Two-way ANOVA. mRNA expression was normalized to *Gapdh*.

Abbreviations: B6, C57BL/6 mice; HFD, high-fat diet; ldSTZ, multiple low dose streptozotocin; Arb. units, arbitrary units.

Supplementary Figure 6. **Generation of the TMEM219$^{flox/flox}$ mouse**.

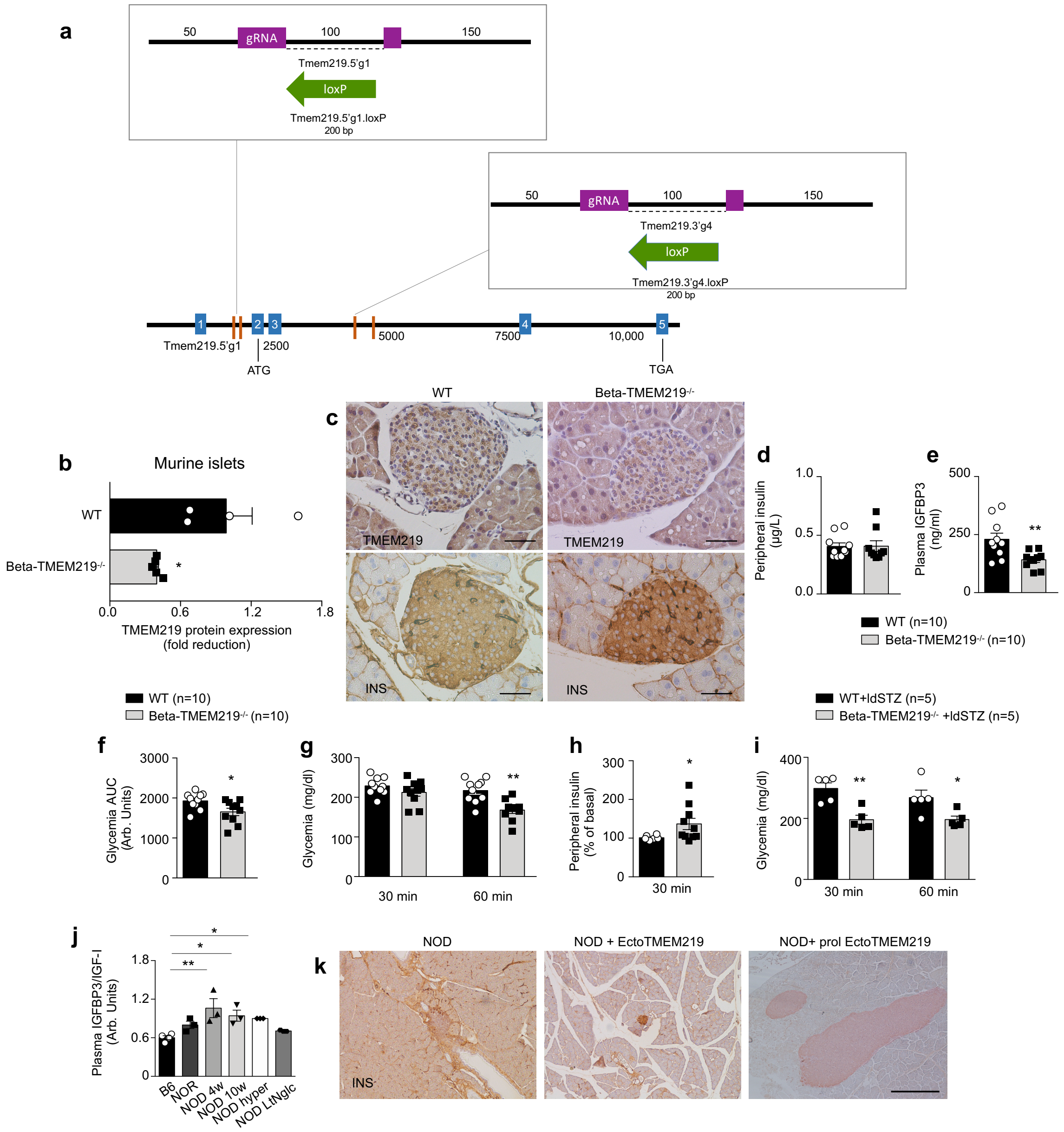

(a). Schematic representation depicting the strategy used to generate the TMEM219$^{flox/flox}$ mouse. **(b)**. TMEM219 protein quantification in dissociated islets obtained from Beta-TMEM219$^{-/-}$ (n=5) and WT mice (n=4). **(c)**. Representative picture of TMEM219 and insulin immunostaining in in WT and in Beta-TMEM219$^{-/-}$. **(d, e)**. Bar graph representing peripheral insulin and IGFBP3 levels measured in Beta-TMEM219$^{-/-}$ and in WT mice (n=8-

10). **(f, g)**. Bar graph showing IPGTT blood glucose AUC (f) and glycemia levels (g) at 30 and 60 minutes in Beta-TMEM219$^{-/-}$ and in WT mice (n=10). **(h)**. Bar graph representing peripheral insulin measured in Beta-TMEM219$^{-/-}$ and in WT mice at 30 minutes of the IPGTT (n=8-10). **(i)**. Bar graph showing IPGTT blood glucose levels measured at 30 and 60 minutes in Beta-TMEM219$^{-/-}$ and in WT mice injected with multiple low-dose streptozotocin (n=5). **(j)**. Bar graphs representing IGFBP3/IGF-I plasma ratio measured in 8-week-old B6 (n=10) mice, in pre-diabetic (4-week and 10-week-old, n=4) and diabetic (hyperglycemic, n=5) NOD mice, in mice resistant to development of autoimmune diabetes (NOR, n=3) and in long-term normoglycemic (LtNglc, n=4) NOD mice. **(k)**. Representative insulin staining in serial pancreatic islet tissue sections obtained from NOD mice treated with ecto-TMEM219 for 10 days or with prolonged ecto-TMEM219, or from untreated NOD mice. 10X original magnification, scale bar, 300 μm. Data are expressed as mean ± standard error of the mean (SEM) unless otherwise reported. *$p<0.05$; **$p<0.01$ by two-sided Mann–Whitney U-test, One-way ANOVA followed by Bonferroni's post-hoc test.

Abbreviations: Beta-TMEM219$^{-/-}$, mice in which TMEM219 was deleted in beta cells; WT, wild type mice in which TMEM219 has not been genetically deleted. NOD, nonobese diabetic mice; LtNglt, long-term normoglycemic NOD mice; NOR, nonobese diabetes-resistant; hyper, hyperglycemic; prol, prolonged ecto-TMEM219 treatment; IPGTT, intraperitoneal glucose tolerance test; Arb. Units, arbitrary units.

Supplementary Figure 7. Characterization of the IGFBP3/TMEM219 binding.

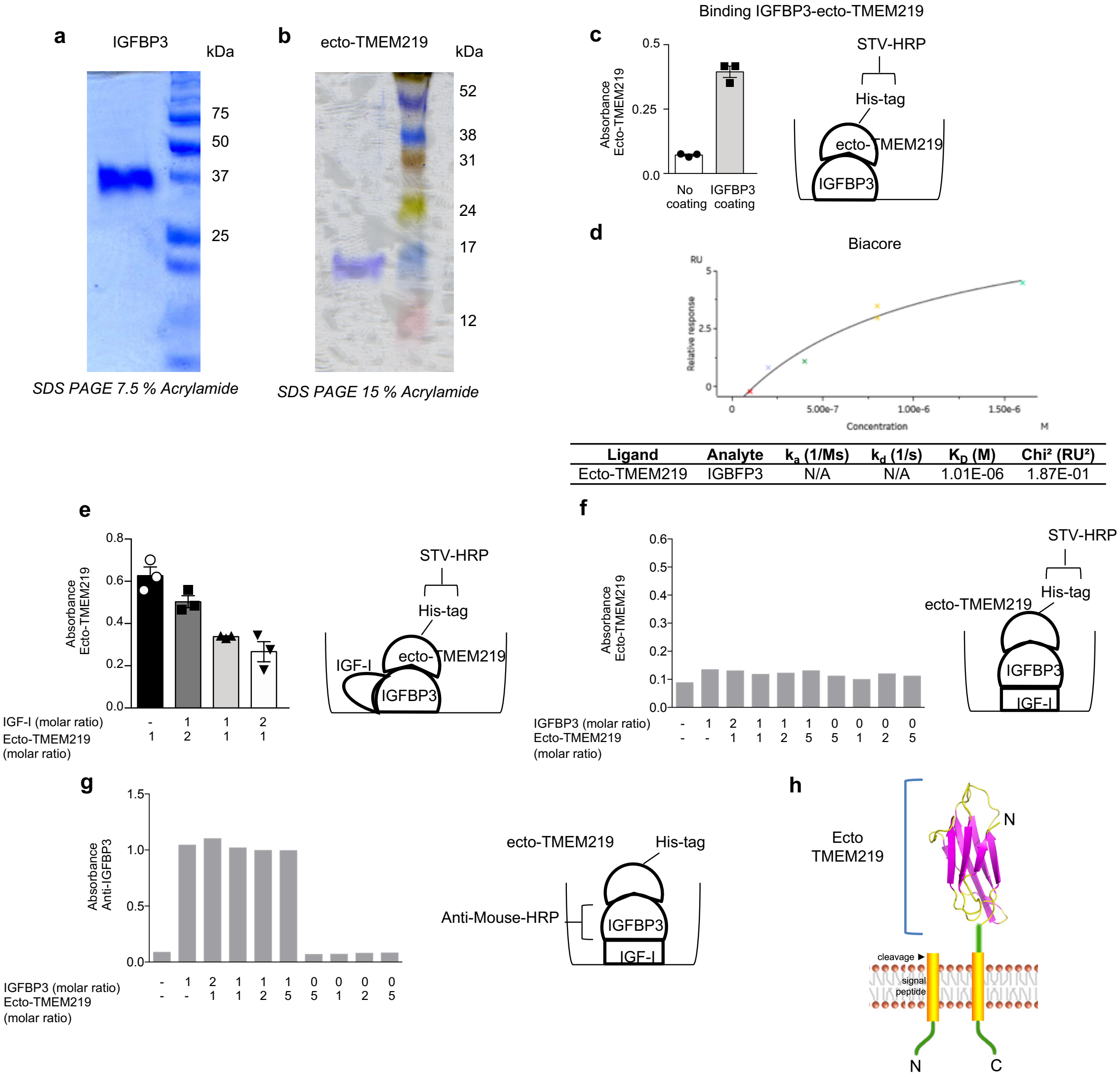

Ligand	Analyte	k_a (1/Ms)	k_d (1/s)	K_D (M)	Chi² (RU²)
Ecto-TMEM219	IGBFP3	N/A	N/A	1.01E-06	1.87E-01

(a, b). Representative gel showing SDS page of recombinant IGFBP3 (R&D, 8874-B3) and ecto-TMEM219 (Genscript). The data shown are representative of two independent experiments. **(c)**. Binding study conducted by ELISA demonstrating that IGFBP3 binds to ecto-TMEM219. Absorbance of ecto-TMEM219 was measured. At least n=3 biologically independent experiments were performed. **(d)**. Measurement of affinity between IGFBP3 and ecto-TMEM219 by using the Biacore 8K. **(e)**. Competitive ELISA binding study showing the preferential binding of IGFBP3 to IGF-I as compared to ecto-TMEM219.

Absorbance of ecto-TMEM219 was measured. At least n=3 biologically independent experiments were performed. **(f, g)**. Competitive ELISA binding study showing that ecto-TMEM219 is unable to displace the IGFBP3/IGF-I binding. Absorbance of ecto-TMEM219 was measured in f and that of anti-IGFBP3 was measured in g. **(h)**. Putative 3D representation of ecto-TMEM219 by using a FFAS-modelled (magenta/yellow) TMEM219 structure in multiple orientations with a transmembrane domain. Data are expressed as mean ± standard error of the mean (SEM) unless otherwise reported.

Abbreviations: STV, streptavidin.

Supplementary References

1. Bianchi C*, et al.* Pathogenetic mechanisms and cardiovascular risk: differences between HbA(1c) and oral glucose tolerance test for the diagnosis of glucose tolerance. *Diabetes Care* **35**, 2607-2612 (2012).

2. Grancini V*, et al.* Central role of the beta-cell in driving regression of diabetes after liver transplantation in cirrhotic patients. *J Hepatol* **70**, 954-962 (2019).

3. D'Addio F*, et al.* Circulating IGF-I and IGFBP3 Levels Control Human Colonic Stem Cell Function and Are Disrupted in Diabetic Enteropathy. *Cell stem cell* **17**, 486-498 (2015).

4. Asfari M, Janjic D, Meda P, Li G, Halban PA, Wollheim CB. Establishment of 2-mercaptoethanol-dependent differentiated insulin-secreting cell lines. *Endocrinology* **130**, 167-178 (1992).

5. Di Rienzo C, Gratton E, Beltram F, Cardarelli F. Fast spatiotemporal correlation spectroscopy to determine protein lateral diffusion laws in live cell membranes. *Proc Natl Acad Sci U S A* **110**, 12307-12312 (2013).

6. Ferri G, Digiacomo L, D'Autilia F, Durso W, Caracciolo G, Cardarelli F. Time-lapse confocal imaging datasets to assess structural and dynamic properties of subcellular nanostructures. *Sci Data* **5**, 180191 (2018).

7. Ferri G*, et al.* Insulin secretory granules labelled with phogrin-fluorescent proteins show alterations in size, mobility and responsiveness to glucose stimulation in living beta-cells. *Sci Rep* **9**, 2890 (2019).

8. Ben Nasr M*, et al.* PD-L1 genetic overexpression or pharmacological restoration in hematopoietic stem and progenitor cells reverses autoimmune diabetes. *Sci Transl Med* **9**, (2017).

9. D'Addio F*, et al.* A novel clinically relevant approach to tip the balance toward regulation in stringent transplant model. *Transplantation* **90**, 260-269 (2010).

10. Rajpathak SN*, et al.* Insulin-like growth factor axis and risk of type 2 diabetes in women. *Diabetes* **61**, 2248-2254 (2012).

11. Bereket A, Lang CH, Blethen SL, Fan J, Frost RA, Wilson TA. Insulin-like growth factor binding protein-3 proteolysis in children with insulin-dependent diabetes mellitus: a possible role for insulin in the regulation of IGFBP-3 protease activity. *J Clin Endocrinol Metab* **80**, 2282-2288 (1995).

12. Drogan D, Schulze MB, Boeing H, Pischon T. Insulin-Like Growth Factor 1 and Insulin-Like Growth Factor-Binding Protein 3 in Relation to the Risk of Type 2 Diabetes Mellitus: Results From the EPIC-Potsdam Study. *Am J Epidemiol* **183**, 553-560 (2016).

13. Aneke-Nash CS*, et al.* Changes in insulin-like growth factor-I and its binding proteins are associated with diabetes mellitus in older adults. *J Am Geriatr Soc* **63**, 902-909 (2015).

14. Peet A*, et al.* Circulating IGF1 and IGFBP3 in relation to the development of beta-cell autoimmunity in young children. *Eur J Endocrinol* **173**, 129-137 (2015).

15. Frystyk J, Skjaerbaek C, Vestbo E, Fisker S, Orskov H. Circulating levels of free insulin-like growth factors in obese subjects: the impact of type 2 diabetes. *Diabetes Metab Res Rev* **15**, 314-322 (1999).

16. Gutefeldt K, Hedman CA, Thyberg ISM, Bachrach-Lindstrom M, Spangeus A, Arnqvist HJ. Dysregulated growth hormone-insulin-like growth factor-1 axis in adult type 1 diabetes with long duration. *Clin Endocrinol (Oxf)*, (2018).

17. Munoz MT, Barrios V, Pozo J, Argente J. Insulin-like growth factor I, its binding proteins 1 and 3, and growth hormone-binding protein in children and adolescents with insulin-dependent diabetes mellitus: clinical implications. *Pediatr Res* **39**, 992-998 (1996).

18. Rajpathak SN*, et al.* Insulin-like growth factor-(IGF)-axis, inflammation, and glucose intolerance among older adults. *Growth Horm IGF Res* **18**, 166-173 (2008).

19. Cinaz P*, et al.* Serum levels of insulin-like growth factor-I and insulin-like growth factor binding protein-3 in children with insulin-dependent diabetes mellitus. *J Pediatr Endocrinol Metab* **9**, 475-482 (1996).

20. Kim MS, Lee DY. Serum insulin-like growth factor-binding protein-3 level correlated with glycemic control and lipid profiles in children and adolescents with type 1 diabetes. *J Pediatr Endocrinol Metab* **27**, 857-861 (2014).

21. Wedrychowicz A, Dziatkowiak H, Nazim J, Sztefko K. Insulin-like growth factor-1 and its binding proteins, IGFBP-1 and IGFBP-3, in adolescents with type-1 diabetes mellitus and microalbuminuria. *Horm Res* **63**, 245-251 (2005).

22. Kim MS, Lee DY. Insulin-like growth factor (IGF)-I and IGF binding proteins axis in diabetes mellitus. *Ann Pediatr Endocrinol Metab* **20**, 69-73 (2015).
