# Supplementary material for: The IGFBP3/TMEM219 pathway regulates beta cell homeostasis
Source: Nat Commun. 2022 Feb 3;13:684. doi: 10.1038/s41467-022-28360-2 (PMC8813914; doi:10.1038/s41467-022-28360-2)
Supplement: Supplementary file 2 — Description of Additional Supplementary Files [file 41467_2022_28360_MOESM2_ESM.pdf]

Description of Additional Supplementary Files

File Name: Supplementary Data 1
Description: List of genes tested in Human Apoptosis PCR Arrays (PAHS-012Z, SABiosciences) and Human Insulin Signaling Pathway PCR Arrays (PAHS-030Z, SABiosciences).

File Name: Supplementary Data 2
Description: List of genes identified by RNAseq analysis. The table reports correspondence between numbers and names of genes detected by RNAseq analysis. Putative surface genes expressed with an RPKM > 2 are shown in Figure 1.
